# Differential Ultra-Wideband Microwave Imaging: Principle Application Challenges

**DOI:** 10.3390/s18072136

**Published:** 2018-07-03

**Authors:** Jürgen Sachs, Sebastian Ley, Thomas Just, Somayyeh Chamaani, Marko Helbig

**Affiliations:** 1 Electronic Measurements and Signal Processing Group, Technische Universität Ilmenau, 98693 Ilmenau, Germany; thomas.just@tu-ilmenau.de (T.J.); somayyeh.chamaani@tu-ilmenau.de (S.C.); 2 Biosignal Processing Group, Technische Universität Ilmenau, 98693 Ilmenau, Germany; sebastian.ley@tu-ilmenau.de (S.L.); marko.helbig@tu-ilmenau.de (M.H.); 3 Faculty of Electrical Engineering, K. N. Toosi University of Technology, 16317 Tehran, Iran

**Keywords:** microwave imaging, medical imaging, vital data capturing, ultra-wideband, target localization, M-sequence, pulse radar, through-wall radar, wooden pest detection

## Abstract

Wideband microwave imaging is of interest wherever optical opaque scenarios need to be analyzed, as these waves can penetrate biological tissues, many building materials, or industrial materials. One of the challenges of microwave imaging is the computation of the image from the measurement data because of the need to solve extensive inverse scattering problems due to the sometimes complicated wave propagation. The inversion problem simplifies if only spatially limited objects—point objects, in the simplest case—with temporally variable scattering properties are of interest. Differential imaging uses this time variance by observing the scenario under test over a certain time interval. Such problems exist in medical diagnostics, in the search for surviving earthquake victims, monitoring of the vitality of persons, detection of wood pests, control of industrial processes, and much more. This paper gives an overview of imaging methods for point-like targets and discusses the impact of target variations onto the radar data. Because the target variations are very weak in many applications, a major issue of differential imaging concerns the suppression of random effects by appropriate data processing and concepts of radar hardware. The paper introduces related methods and approaches, and some applications illustrate their performance.

## 1. Introduction

**Figure 1:**
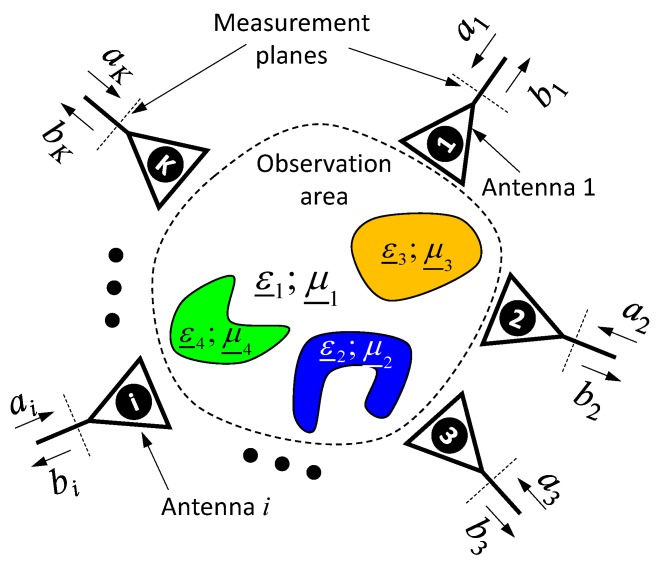
Generic microwave imaging setup. Note that in some applications, the antennas may also be located inside the observation area.

Microwave imaging may be considered as a method to reconstruct the spatial distribution of matter (bodies, objects) based on the electromagnetic sounding of an observation space of interest. Figure 1 demonstrates the principle. The observation space may be completely or partially surrounded by antennas. The antennas emit electromagnetic fields into the observation space and receive the fields scattered at the surface of the bodies due to the different propagation parameters at both sides of the boundaries. Such propagation parameters are either the permittivity $\underline{\varepsilon }$ and the permeability $\underline{\mu }$ of the involved substances or the propagation speed $\underline{c}=1/\sqrt{\underline{\varepsilon }\;\underline{\mu }}$ and the intrinsic impedance $\underline{Z}=\sqrt{\underline{\mu }/\underline{\varepsilon }}$. Typically, every substance is characterized by a specific set of such parameters, which makes different substances distinguishable by electromagnetic sounding.

Obviously, by referring to Figure 1, one can measure all combinations between the antenna feeding signals $a_{i};\;i=1\cdots K$ and the received signals $b_{j};\;j=1\cdots K$, where the received signals are affected by the inner structure of the observation space. Hence, from the measurement, we get a set of response functions $S_{ji};\;i,j=1\cdots K$

(1) $$\left[\begin{array}{c} {\underline{b}}_{1}\\ {\underline{b}}_{2}\\ \vdots \\ {\underline{b}}_{K} \end{array}\right]=\left[\begin{array}{cccc} {\underline{S}}_{11} & {\underline{S}}_{12} & \cdots & {\underline{S}}_{1K}\\ {\underline{S}}_{21} & {\underline{S}}_{22} & \cdots & {\underline{S}}_{2K}\\ \vdots & \vdots & \ddots & \vdots \\ {\underline{S}}_{K1} & {\underline{S}}_{K2} & \cdots & {\underline{S}}_{KK} \end{array}\right]\cdot \left[\begin{array}{c} {\underline{a}}_{1}\\ {\underline{a}}_{2}\\ \vdots \\ {\underline{a}}_{K} \end{array}\right]\;\to \;\underline{b}\left(f\right)=\underline{S}\left(f\right)\;\underline{a}\left(f\right)$$

or

$$\left[\begin{array}{c} b_{1}\\ b_{2}\\ \vdots \\ b_{K} \end{array}\right]=\left[\begin{array}{cccc} S_{11} & S_{12} & \cdots & S_{1K}\\ S_{21} & S_{22} & \cdots & S_{2K}\\ \vdots & \vdots & \ddots & \vdots \\ S_{K1} & S_{K2} & \cdots & S_{KK} \end{array}\right]*\left[\begin{array}{c} a_{1}\\ a_{2}\\ \vdots \\ a_{K} \end{array}\right]\;\to \;b\left(t\right)=S\left(t\right)*a\left(t\right),$$

which carry the information (encoded by Maxwell’s equations and the boundary conditions) about the observation space of interest. Note that the matrices of the response functions are symmetric due to reciprocity (i.e., $\underline{S}={\underline{S}}^{T}\;\mathrm{or}\;S=S^{T}$, where ${}^{T}$ means transpose). The underscored quantities in the upper equation refer to complex valued frequency functions, which are gained from sinewave measurements. The lower equation deals with real valued time functions resulting from measurements with arbitrary-shaped ultra-wideband (UWB) signals, where the symbol ∗ means convolution [1]. Finally, the aim of the imaging procedure is to “decode” the internal structure of the observation space of interest from the measured quantities. For that purpose, the equations describing wave propagation and scattering have to be inverted, using a method called “inverse scattering”. A comprehensive introduction in this technique and an example for ground penetrating radar are, for example, given in [2,3]. The solution of inverse problems is typically ill-conditioned and numerically quite expensive. Hence, one relies in many cases on some prior knowledge about the observation space of interest and on simplifications of the inversion procedure (e.g., by omitting multipath propagation (often assigned as Born approximation), which is however only applicable in low contrast scenarios).

Furthermore, one should note that the material parameters $\underline{\varepsilon },\;\underline{\mu }$ or $\underline{Z},\;\underline{c}$ are typically frequency-dependent, and they may be complex valued (assigned by underscore character) with the imaginary part related to propagation losses. If these losses are too large in the scenario under test (SUT), the observation space becomes opaque, and we will not be able to explore its inner structure. Fortunately, most substances (with the exception of metals) are more or less transparent for electromagnetic waves within the microwave frequencies, which makes this imaging method attractive for analyzing the internal structure of optically opaque objects.

The image resolution (i.e., the ability to separate two small, identical, and closely located objects) depends basically on a spatial parameter of the sounding wave (i.e., wavelength in the case of sinewaves or pulse length and coherence length, respectively, in the cases of short-pulse or UWB-spread spectrum signals), and the solid angle under which the objects are illuminated and observed [1]. The last point is particularly interesting if the test area cannot be completely surrounded by the antennas, which is often the case in practice. From this observation, it becomes clear that microwave images will never have a resolution as good as optical images due to the much larger wavelength or coherence length of these waves, and it becomes also obvious that the sounding waves should operate at frequencies as high as possible. However, the upper frequency bound is often limited by the rising propagation losses with increasing frequency. In many applications, the water content of the different substances (e.g., biological tissue or soil) represents a limiting factor, because water induces losses rapidly increasing beyond 2 GHz. Newer discussions on that topic [4] suspect a usable bandwidth of up to 40 GHz, which will be, however, quite challenging to implement in practice for penetration depths larger than 1 cm.

A further important feature of image quality is the contrast that represents the ratio between the “brightest” and “darkest” image pixels. Bright points are linked to strong reflections caused by extended boundaries (i.e., large bodies) with large gradients of permittivity or permeability. Dark image pixels correspond to weak scattering objects, which are typically small and/or have low permittivity/permeability contrast with respect to their environment. The achievable contrast depends on many factors:

- the sidelobes of the point-spread function of the imaging system, which are a matter of the antenna array structure, the number of antennas, and the bandwidth of the received signals [1,5];
- multipath signals caused by scattering at dominant objects overpowering the reflections of weak bodies;
- device internal clutter, caused by imperfections of the measurement equipment (which can only be suppressed up to a certain degree by appropriate device calibration);
- time extension of the sounding waves due to the limited decay rate of the antenna impulse response; and
- receiver noise, propagation loss, and others.

This basically prevents the detection or analysis of weak scattering objects by microwave sensing if they are embedded in a strong, multipath environment.

However, there is an exception if the weak scattering target of interest is subjected to some variations, which may be caused by the following:

- temporal fluctuations inherently connected with the test scenario (e.g., the vital motion of inner organs of humans and animals, the breathing motion of buried survivors after an earthquake, and the motion of wood-destroying insects, as well as slowly running events, such as the putrefaction of biological substances, the healing process after a medical surgery [6], post-event monitoring of stroke [7], and many more);
- a targeted influence of the hidden object of interest via modification of its position in space, its volume, and its permittivity or permeability (e.g., the targeting of malignant tissue by nanoparticles, permittivity variation by local heating or cooling [8,9], water accumulation in hygroscopic substances, etc.); and
- small deviations between two largely identical SUTs (cancer in one of the two female breasts [10], foreign objects in chocolate, other identical food pieces, etc.).

In all of these examples, we are only interested in the imaging and localization, respectively, of small differences—hence, we call it differential imaging—in the scattering scenario in its different states. Basically, the imaging may be based on a tomographic approach (see e.g., [9]) or on radar image processing. Tomographic methods usually work with narrowband signals and require a dense antenna array, while radar-based techniques need wideband sounding signals but do not require a dense antenna array [5]. The aim of the paper is to introduce the reader to the concept of differential imaging and to familiarize him or her with the experimental challenges of a successful data capturing and target extraction. For the sake of introduction, we assume here that the objects of interest are relatively small compared to the size of the observation space. This allows us to deal with the simpler radar imaging approach instead of the inverse scattering applied in microwave tomography. The experimental challenges will, however, not be touched by that simplification.

In what follows, a simplified signal model to describe the scattering scenario will be introduced with emphasis on the most important aspects of differential imaging. Based on this consideration the most important parameters of UWB devices for differential imaging will be considered. Several examples and measurements are shown for illustration.

## 2. Signal Model for Small Time-Variant Scattering Objects

By restricting ourselves to small objects of interest, which are sufficiently far from the antennas, the set of differential equations (i.e., Maxwell equations) describing the scenario may be approximated by a simple transmission model. It will be finally based on the Friis transmission formula and the radar equation, which we extend to time-domain conditions here. In order to make that approximation already valid for short distances, the involved antennas should be quite small.

In what follows, we first introduce this transmission model for the simple case of free space propagation and summarize some methods of localizing/imaging of small, invariant objects without the need of an inversion of Maxwell’s equations. We then discuss variant objects under free space conditions and some methods are introduced to detect them under noisy conditions. For the sake of brevity, we will only refer in this connection to the time variance of those objects. But, this approach will also include the “targeted object modification” (which is typically time-dependent) and the “difference between two scenarios”-method, if we regard the related measurements as sequentially done in (observation) time T. Finally, we will extend the scenario model to strong multipath conditions (i.e., the case will be considered in which a weak time-variant object is embedded in a strong multipath environment).

### 2.1. Invariant Object in Free Space and Its Localization

**Figure 2:**
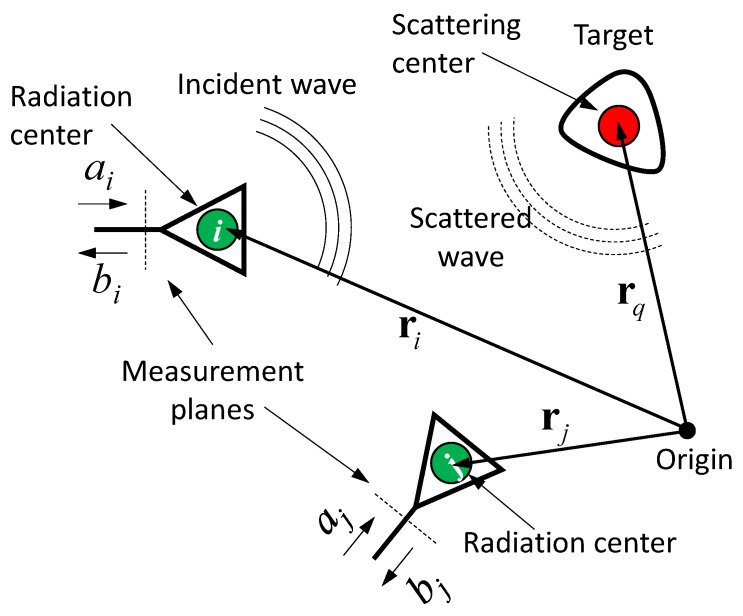
Arbitrary distributed antenna array with single target in free space.

The generic situation is depicted in Figure 2. The antennas and target are referred to as points in space assigned by their position vectors $r_{i}$ and $r_{q}$. We call these points radiation and scattering centers, respectively. The interaction of the involved objects—the antennas and target—with the electric field, we describe by impulse response functions (IRF) $T_{i}\left(t\right)$, $R_{i}\left(t\right)$ and $\Gamma_{i}\left(t\right)$, Λ(t). Herein, t refers to the propagation time (often also called fast time). $T_{i}\left(t\right)$ and $R_{i}\left(t\right)$ are the transmission IRFs of antenna i if it works in transmitter and receiver mode, respectively. Both are linked via the reciprocity relation $T_{i}\left(t\right)=\frac{1}{2\pi c}\frac{d}{dt}R_{i}\left(t\right)$, where c is the speed of light in the propagation medium. $\Gamma_{i}\left(t\right)$ and Λ(t) represent reflection IRFs of the antenna feeding and the target. For an introduction into the concept of impulse response functions see [1]. Any angular dependencies and polarimetric issues of $T_{i},\;R_{i}$ and Λ, as well as the aperture reflection of the antenna, we will omit here for the sake of brevity. For such a scenario, the individual response functions in (1) can be expressed by the time domain Friis-formula and radar equation by (δ(t)—Dirac delta function):

(2) $$\begin{array}{ccc} S_{ii}\left(t\right) & = & \Gamma_{i}\left(t\right)*\delta \left(t-2\tau_{a}\right)+\frac{1}{r_{iq}^{2}}T_{i}\left(t\right)*\Lambda \left(t\right)*R_{i}\left(t\right)*\delta \left(t-2\left(\tau_{iq}+\tau_{a}\right)\right)\\ S_{ji}\left(t\right) & = & \frac{1}{r_{ji}\;}T_{j}\left(t\right)*R_{i}\left(t\right)*\delta \left(t-\left(\tau_{ji}+2\tau_{a}\right)\right)+\frac{1}{r_{iq}\;r_{jq}}T_{j}\left(t\right)*\Lambda \left(t\right)*R_{i}\left(t\right)*\delta \left(t-\left(\tau_{iq}+\tau_{jq}+2\tau_{a}\right)\right)\\ \tau_{iq} & = & \frac{r_{iq}}{c};\;r_{iq}=\left|r_{q}-r_{i}\right|;\;\tau_{ji}=\frac{r_{ji}}{c};\;r_{ji}=\left|r_{i}-r_{j}\right|. \end{array}$$

Herein, the left term of $S_{ii}$ represents the feed point reflections of the antenna, and the right term is a mono-static radar equation, while $S_{ji}$ is derived from the Friis-formula (left term; often called crosstalk) and a bi-static radar equation (right term). Multiple reflections between antenna–antenna and antennas–target have been omitted in Equation (2). The delay times $\tau_{a},\;\tau_{ji},\;\tau_{iq}$ refer to the propagation delay between the measurement plan and the radiation center of the antennas, the propagation time between two antennas, and the propagation time between the antenna and the target, respectively.

Following (2), the response matrix S can be decomposed into two parts as follows:

(3) $$S=S_{0}+S_{\Lambda },$$

where $S_{0}$ only involves antenna effects (i.e., feed point reflection and crosstalk) and $S_{\Lambda }$ is called the multi-static response matrix, which covers all transmission paths including the scattering object.

**Figure 3:**
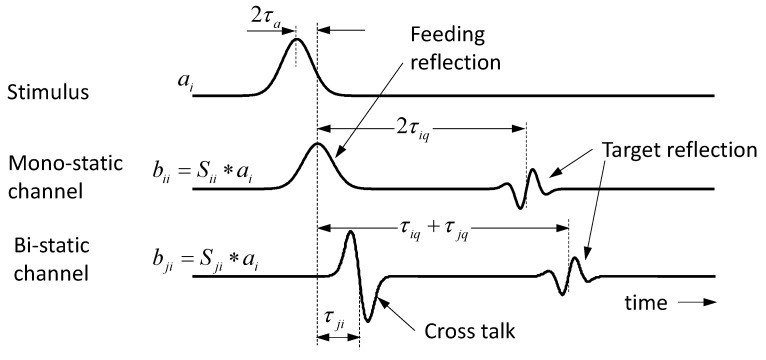
Typical signals for an idealized scenario with electrically small antennas and a point scatterer. Antenna i is stimulated with a Gaussian pulse, while all other antennas only act as receiver $a_{j}=0;\;j\neq i$.

Figure 3 illustrates typical signals for an idealized scenario with electrically short antennas and a point scatterer. Note that the antenna IRF in transmission and receiving mode is simply the derivative $T_{i}∼\frac{d}{dt}\cdots$ and a delta function $R_{j}\left(t\right)∼\delta \left(t\right)$, respectively. The IRF of a point scatterer leads to a second derivative $\Lambda \left(t\right)∼\frac{d^{2}}{dt^{2}}\cdots$ [1]. The signal $b_{ji}$ refers to the received signal of antenna j, if antenna i is stimulated by the pulse. 

For the reconstruction of the target locations (i.e., the image of the scenario) from the measurements $b_{ji}$, one has several options.

*Method 1*: The volume to be observed is subdivided into a grid of voxels. In order to get the intensity value $I_{p}$ of the voxel located at position $r_{v}$, one superimposes the signal components received by all antennas with propagation times corresponding to the related voxel-antenna distance. Note, for that purpose, the wave speed is supposed to be known. In a mathematically generalized form, inspired from the p-norm, this may be expressed in different ways, as for example by (p=0,1,2,3,⋯ ):

(4) $$\begin{array}{ccc} I_{1,p}\left(r_{v}\right) & = & \sqrt[{p}]{\int_{-\infty }^{\infty }w\left(t\right)\left|\sum_{i=1}^{K}\sum_{j=1}^{K}{\left(h_{ji}\left(t\right)*b_{ji}\left(t+\tau_{v}\right)\right)}^{p}\right|dt}\\ I_{2,p}\left(r_{v}\right) & = & \sqrt[{p}]{\int_{-\infty }^{\infty }w\left(t\right)\sum_{i=1}^{K}\sum_{j=1}^{K}{\left|h_{ji}\left(t\right)*b_{ji}\left(t+\tau_{v}\right)\right|}^{p}dt}\\ \tau_{v} & = & \frac{\left|r_{i}-r_{v}\right|+\left|r_{j}-r_{v}\right|}{c}+2\tau_{a}. \end{array}$$

If the voxel position coincides with the target position $r_{v}=r_{q}$, the signals are coherently superimposed, leading to a large intensity value. In the opposite case (i.e., $r_{v}\neq r_{q}$), the signals are incoherently added, so the voxel intensity tends to small values. For the suppression of measurement errors, sidelobes, or other image defects, it may be meaningful to modify every signal $b_{ji}\left(t\right)$ by an individual weighting function $h_{ji}\left(t\right)$ before summing (see e.g., [11,12,13,14,15] for examples). Furthermore, in Equation (4), w(t) represents a gating function whose duration is on the order of the width of the stimulus pulse. This ensures that only the desired signal sections are added up. In Equation (4), for p, typically 1 or 2 are also assigned as a delay-and-sum approach, which is widely used in the literature.

**Figure 4:**
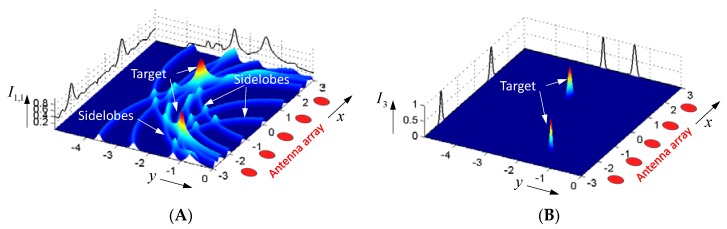
Intensity plot of a two-dimensional (2D)-scenario with two point scatterers of equal reflectivity. (**A**) Delay-and-Sum approach. (**B**) Delay-and-Multiply method.

Figure 4A illustrates an intensity plot based on the delay-and-sum approach for a simple two-dimensional (2D)-scenario with a five-element linear antenna array. Obviously, the method provokes many sidelobes, which limit the contrast of the image. To improve the contrast, the number of antennas has to be increased or other methods of signal superposition have to be applied. Equations (5) and (6) depict two examples. The first method, we call the delay-and-multiply approach. A related result is demonstrated in Figure 4B. Obviously, the contrast is dramatically increased, because the sidelobes have disappeared. Nevertheless, the method needs some care, because an unwanted zero in the data, which actually should be superimposed, will “destroy” the related target.

(5) $$I_{3}\left(r_{v}\right)=\int_{-\infty }^{\infty }w\left(t\right)\prod_{i}\prod_{j}\left|h_{ji}\left(t\right)*b_{ji}\left(t+\tau_{v}\right)\right|dt\;.$$

Finally, the routine Equation (6) superimposes a set of cross-correlation functions—also assigned as delay-multiply-and-sum—determined from the captured response signals [16,17,18,19,20].

(6) $$\begin{array}{l} I_{4}\left(r_{v}\right)=\left|\int_{-\infty }^{\infty }w\left(t\right)\sum_{\begin{array}{l} i=1\\ i\neq k,l \end{array}}^{K}\sum_{\begin{array}{l} j=1\\ j\neq k,l \end{array}}^{K}\sum_{k=1}^{K}\sum_{l=1}^{K}h_{jikl}\left(t\right)*b_{ji}\left(t+\tau_{v}^{\left(ji\right)}\right)*b_{lk}\left(-t-\tau_{v}^{\left(lk\right)}\right)dt\right|\\ \tau_{v}^{\left(ji\right)}=\frac{\left|r_{i}-r_{v}\right|+\left|r_{j}-r_{v}\right|}{c}+2\tau_{a};\;\tau_{v}^{\left(lk\right)}=\frac{\left|r_{k}-r_{v}\right|+\left|r_{l}-r_{v}\right|}{c}+2\tau_{a}. \end{array}$$

*Method 2*: Instead of superimposing the measured signals as demonstrated in Equations (4)–(6), one may also try to numerically solve the problem of target localization. If we consider a single measurement $b_{ji}\left(t\right)$ (compare Figure 3) or even a cross-correlation function between signals $b_{ji}\left(t\right)$ and $b_{lk}\left(t\right)$, we can identify the propagation time of arrival (ToA) and the propagation time difference of arrival (TDoA), respectively, between the involved antennas and the target. Based on the known wave speed, the propagation distance may be estimated, so a quadric surface (sphere, ellipsoid, elliptic hyperboloid) can be calculated on which the target is localized. By repeating this for different antenna positions, the intersections of all quadric surfaces finally gives the actual target position.

In what follows, the localization of a single target shall be illustrated. For the sake of demonstration, we restrict ourselves to mono-static measurements $b_{ii}\left(t\right)$ solely, which provide us the target range via roundtrip time measurement $r_{iq}=\tau_{iq}c/2$ (refer to Figure 3). Corresponding to the measurement setup in Figure 2, one finds:

(7) $$r_{iq}^{2}={\left(r_{q}-r_{i}\right)}^{T}\left(r_{q}-r_{i}\right)=r_{q}{}^{2}+r_{i}^{2}-2r_{i}^{T}r_{q},$$

where $r_{i}$ and $r_{q}$ represent [3,1] position vectors. In order to remove $r_{q}^{2}$ from the equation, the results from two different antennas are subtracted. For an antenna array of K≥4 antennas, this leads to a set of P=K!/(2(K−2)!)≥6 combinations, which are arranged in following manner:

(8) $$\left[\begin{array}{c} {\left(r_{1}-r_{2}\right)}^{T}\\ {\left(r_{1}-r_{3}\right)}^{T}\\ \vdots \\ {\left(r_{i}-r_{j}\right)}^{T}\\ \vdots \end{array}\right]\;r_{q}=\frac{1}{2}\left[\begin{array}{c} \left(r_{1}^{2}-r_{2}^{2}\right)-\left(r_{1q}^{2}-r_{2q}^{2}\right)\\ \left(r_{1}^{2}-r_{3}^{2}\right)-\left(r_{1q}^{2}-r_{3q}^{2}\right)\\ \vdots \\ \left(r_{i}^{2}-r_{j}^{2}\right)-\left(r_{iq}^{2}-r_{jq}^{2}\right)\\ \vdots \end{array}\right]\;\;=R\;r_{q}=\frac{1}{2}Q.$$

Here, R represents a [P,3] matrix of the known antenna position vectors, $r_{q}$ is the [3,1] column vector of the wanted target position, and Q is a [P,1] column vector built from known antenna distances and the measured target distances. The minimum least square solution of the overdetermined Equation (8) for the target position is given by the following:

(9) $$r_{q}=\frac{1}{2}{\left(R^{T}R\right)}^{-1}R^{T}Q.$$

This type of solution is optimal if the measurement errors of the involved quantities are Gaussian distributed. The squaring of $r_{iq}$ in Equation (8) leads, however, to a non-Gaussian error distribution, and Equation (9) will then provide a biased solution.

In order to avoid the bias, a maximum likelihood approach can be applied, which aims to maximize the probability density function (PDF) in Equation (10) with respect to the target position $r_{q}$:

(10) $$p\left(r_{v}|D\right)=\frac{e^{-\frac{1}{2}{\left(D_{v}-D\right)}^{T}\Sigma^{-1}\left(D_{v}-D\right)}}{\sqrt{\det\left(2\pi \Sigma \right)}}\;\Rightarrow \;r_{q}=\underset{r_{v}}{\arg\max}\left\{p\left(r_{v}|D\right)\right\}=\underset{r_{v}}{\arg\min}\left\{{\left(D_{v}-D\right)}^{T}\Sigma^{-1}\left(D_{v}-D\right)\right\}.$$

Here again, one subdivides the volume to be observed in voxels assigned by their position $r_{v}$. The distances of a voxel to all antenna positions are summarized in the [1,K] column vector $D_{v}={\left[\begin{array}{cccc} \left|r_{1}-r_{v}\right| & \left|r_{2}-r_{v}\right| & \cdots & \left|r_{K}-r_{v}\right| \end{array}\right]}^{T}$. The actual distances between the antennas and the target gained from roundtrip time measurements, we have arranged in the [1,K] column vector $D={\left[\begin{array}{cccc} r_{1q} & r_{2q} & \;\cdots & r_{Kq} \end{array}\right]}^{T}$, and the uncertainties of the measurement are represented by the [K,K] covariance matrix $\Sigma \approx \sigma^{2}I$, which is a diagonal matrix if the range measurements are mutually independent. I is the identity matrix, and $\sigma^{2}$ gives the variance of the range estimation, which can be taken often as equal for all measurement channels.

**Figure 5:**
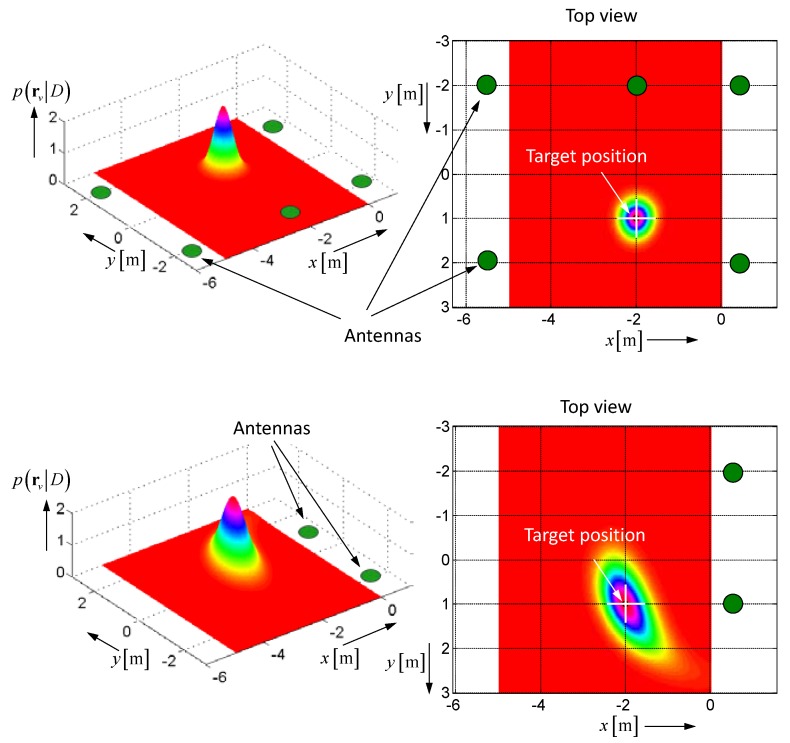
Simulated example of the probability density function (PDF) for a 2D-scenario. The standard deviation of the measurements was assumed to be σ=0.3 m. (**Top**) the observation area is surrounded by the antenna. (**Bottom**) the observation area is illuminated only from one side.

Figure 5 depicts a simple 2D-example of the PDF illustrating the impact of the measurement uncertainty σ and the array structure onto the target localization. In this context, it should not go unmentioned that the measurement uncertainty is not only due to random measurement errors, but also caused by the ambiguity of the distance definition of spatially extended objects [21,22]. Furthermore, the method is only able to estimate the position of a single target, so in multi-target scenarios, the roundtrip times extracted from the measurement data must be correctly assigned to the related targets beforehand.

*Method 3*: The last imaging approach, at least that we will mention here, exploits the reciprocity of the transmission paths characterized by the multi-static response matrix $S_{\Lambda }$. Further it is limited to a small number of point scatterers involved in the test scenario; hence, the method is also assigned as sparse scene imaging. For details of the method, the interested reader is referred to [1,23,24,25,26,27]. For the first trial, the reciprocity properties of $S_{\Lambda }$ were used by the DORT (Decomposition de l'Operateur de Retournement Temporel) approach [28] with the aim of concentrating wave energy at the position of the strongest scatterer under the multi-path condition. Related ideas may also be adapted for imaging purposes, which exploit Eigen-value or singular value decompositions and the MUSIC concept for target localization. It should be noted that this method assumes knowledge of the wave propagation speed within the scenario of the test.

### 2.2. Time-Variant Objects and Their Emphasis from Noise

Restricting ourselves only to the multi-static transmission matrix, its components may be expressed as follows in the case of a time-variant scenario (compare also Equation (2)):

(11) $$S_{\Lambda ,ji}\left(t,T\right)=\frac{1}{r_{iq}\left(T\right)\;r_{jq}\left(T\right)}\Theta \left(t\right)*\Lambda \left(t,T\right)*\delta \left(t-\left(\frac{r_{iq}\left(T\right)\;+r_{jq}\left(T\right)}{c}\right)\right).$$

Here, T symbolizes the observation (slow) time. Assuming antennas at fixed positions, the time variance can only affect the target reflectivity Λ(t,T) and the target ranges $r_{iq}\left(T\right),\;r_{jq}\left(T\right)$. Observation time-independent components in (11) are summarized by $\Theta \left(t\right)=T_{j}\left(t\right)*R_{i}\left(t\right)*\delta \left(t-2\tau_{a}\right)$.

**Figure 6:**
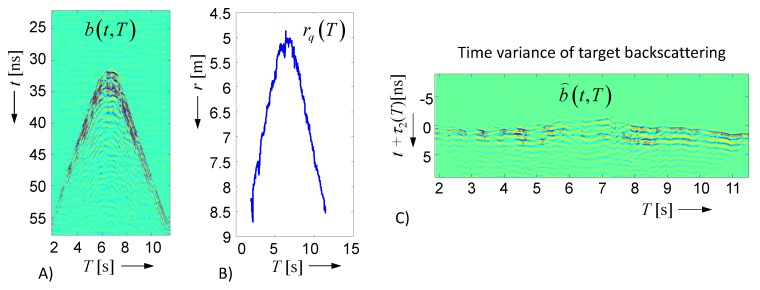
Data of a walking person measured from a mono-static radar. (**A**) radargram; (**B**) target range; and (**C**) time variance of target backscattering.

In order to capture the time variance of the scenario, it is measured in typically regular time intervals ΔT, and the measurements are displayed as functions of the observation time T (i.e., b(t,T)=S(t,T)∗a(t)). The related representation is called a radargram, as exemplified in Figure 6A for a walking person, which moves toward the radar antennas and back. From these measurements, the roundtrip time $\tau_{2}$ has to be estimated. This is not a task of an unambiguous result, because the time shape of the back scattered signal permanently changes (refer Figure 6C). As a consequence, the precision of the target position does not only depend on the range resolution of the UWB radar, but also on the variability of the target response, which is a matter of geometric shape variations during walking. In this paper, as a kind of compromise, the energetic center $\tau_{2}$ is used to estimate the target distance as follows:

(12) $$\begin{array}{l} \tau_{2}^{2}\left(T\right)=\frac{\int t\;b^{2}\left(t,T\right)dt}{\int b^{2}\left(t,T\right)dt}\\ r_{q}\left(T\right)=\frac{\tau_{2}\left(T\right)\;c}{2}. \end{array}$$

By removing the range influence from the radar data via Equation (13) below, one actually may observe the variability of the backscattering while walking (Figure 6C).

(13) $$\overset{⌢}{b}\left(t,T\right)=r_{q}^{2}b\left(t+\tau_{2}\left(T\right),T\right).$$

In order to image the target motion in space, one can follow one of the approaches discussed in Section 2.1 for every timepoint of the observation time.

In many applications, the targets are small, weakly reflecting and moving only a little. This issue, we will address below. A small-sized target leads to Rayleigh-scattering, which provokes a twofold differentiation of the incident field. Hence, in the case of a time variance, such a target may only vary the strength of the backscattering caused by variations of its permittivity, permeability, or volume. This, we will express by:

(14) $$\Lambda \left(t,T\right)=\left(\Lambda_{0}+\Delta \Lambda \left(T\right)\right)\frac{d^{2}}{dt^{2}}\cdots$$

Here, $\Lambda_{0}$ represents the average reflection strength of the target, and ΔΛ symbolizes its variable part. If the target finally also moves a little by Δr, the receiver signals can be modeled by (15). Without loss of generality, we only refer to the mono-static components here. Joining the observation time-independent part Θ(t) with the twofold derivative in Equation (14) $\Phi \left(t\right)=d^{2}\left(\Theta \left(t\right)*a\left(t\right)\right)/dt^{2}$ and insertion of Equation (14) in Equation (11), the receiving signal becomes as follows:

(15) $$\begin{array}{c} b_{ii}\left(t,T\right)=\frac{\Lambda_{0}+\Delta \Lambda \left(T\right)}{{\left(r_{iq,0}+\Delta r_{iq}\left(T\right)\right)}^{2}\;}\Phi \left(t\right)*\delta \left(t-\frac{2\left(r_{iq,0}+\Delta r_{iq}\left(T\right)\right)}{c}\right)+{\underset{˜}{\nu }}_{ii}\left(t,T\right)\\ =\frac{\Lambda_{0}+\Delta \Lambda \left(T\right)}{{\left(r_{iq,0}+\Delta r_{iq}\left(T\right)\right)}^{2}\;}\Phi \left(\xi -\frac{2\Delta r_{iq}\left(T\right)}{c}\right)+{\underset{˜}{\nu }}_{ii}\left(t,T\right)\;. \end{array}$$

To shorten the notation, we include the delay term in the argument of Φ and substitute $\xi =t-2r_{iq,0}/c$, where $r_{iq,0}=r_{iq}\left(T=0\right)$ is the range of the target at the beginning of the observation time. In order to approach realistic conditions, we have also added receiver noise now, which is modeled by the random process ${\underset{˜}{\nu }}_{ii}\left(t,T\right)$. Developing Equation (15) in a Taylor series and omitting higher terms and cross-terms yields the following (using $\dot{\Phi }=d\Phi /dt$):

(16) $$\begin{array}{c} b_{ii}\left(t,T\right)\approx \frac{\Lambda_{0}}{r_{iq,0}}[\underset{A}{\underset{︸}{\Phi \left(\xi \right)}}+\underset{B}{\underset{︸}{\left(\frac{\Delta \Lambda \left(T\right)}{\Lambda_{0}}-\frac{\Delta r_{iq}\left(T\right)}{r_{iq,0}}\right)}}\Phi \left(\xi \right)+\underset{C}{\underset{︸}{\frac{2}{c}\dot{\Phi }\left(\xi \right)\Delta r_{iq}\left(T\right)}}+\\ +\underset{D}{\underset{︸}{\frac{2}{c^{2}}\ddot{\Phi }\left(\xi \right){\left(\Delta r_{iq}\left(T\right)\right)}^{2}}}+\cdots ]+{\underset{˜}{\nu }}_{ii}\left(t,T\right)\;. \end{array}$$

**Figure 7:**
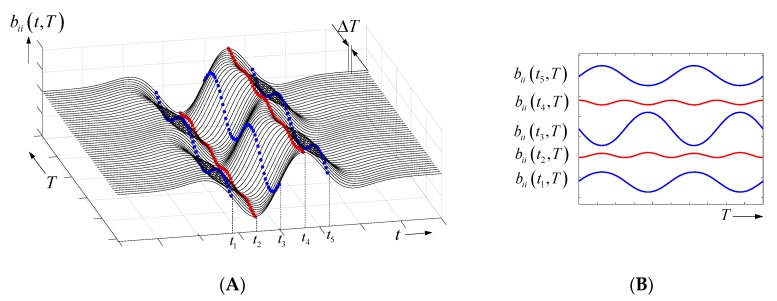
Weakly sinusoidal moving point scatterer. (**A**) Section of the time-variant receiving signal for the noise-free case. The variation of some data samples captured at propagation time $t_{1}\cdots t_{5}$ is emphasized. (**B**) Time variance of the selected data samples (DC-value ignored).

As seen from Equation (16), the radar response $b_{ii}\left(t,T\right)$ is composed from several components. Term A represents the observation time-independent part (i.e., the response of the target in a static state). Term B is an amplitude modulation of the channel response, where the second term $\Delta r_{iq}/r_{iq,0}$ is usually negligible compared to the effect in terms C and D. Terms C and D are caused from time-delay modulations. The modulation term C is dominant at the signal edges (large first derivative) of the received signals. It is proportional to the target motion $\Delta r_{iq}\left(T\right)$. The modulation term D mainly appears in the region of peak values of the receiving signal, because there the magnitude of the second derivative is maximum. This term is usually of minor interest, because it is less sensitive than term C and it provides only the squared target motion. Figure 7 illustrates both terms for a weak sinusoidal target motion. Figure 7A picks out the signal section, which is located around the target reflection (compare Figure 3 for the complete signal). In practice, the received signal is sampled. Hence, we know it only at discrete timepoints. Five such sampling points $t_{1}\cdots t_{5}$ are selected, and their variation in observation time is emphasized. Two points at $t_{1},\;t_{5}$ are placed on a falling signal edge, one point at $t_{3}$ is located at the rising edge, and two points at $t_{2},\;t_{4}$ are to be found close to signal peaks. Figure 7B plots the observation time variation of the different samples. One can observe the following:

- the largest modulation is provided by the sample located at the steepest part of the received signal;
- the modulations at rising and falling edges are inverted; and
- the modulation at the signal peaks has double frequency (due to the squaring) and is quite weak.

For motionless targets, where only its reflectivity ΔΛ is time variant, the modulation term B is responsible. It provides the largest contribution at the signal peaks. 

In the case of properly designed measurement receivers, which are typically based on sub-sampling, the receiver noise ${\underset{˜}{\nu }}_{ij}\left(t,T\right)$ of the different measurements may be considered as Gaussian distributed with a white spectrum in both t and T, as well as uncorrelated between different response functions and measurement channels. Moreover, as will be seen in the next section, the strength of the noise will depend on the received signal itself, and its variance becomes dependent on propagation time:

(17) $$\mathrm{var}\left\{\nu_{ii}\left(t,T\right)\right\}=\sigma_{v}^{2}\left(t\right)=\sigma_{n}^{2}+{\left({\dot{b}}_{ii}\left(t\right)\right)}^{2}\;\varphi_{j}^{2},$$

(i.e., the noise increases at steep signal edges). Here, $\sigma_{n}^{2}$ characterizes the variance of the additive random effect as thermal and quantization noise, while $\varphi_{j}^{2}$ refers to the variance of the sampling jitter, which can be considered as “time noise”. These properties of the raw (i.e., unfiltered) data for mono- and bi-static channels are summarized in the following (Gaussian distributed; white; uncorrelated):

(18) $$\begin{array}{c} {\underset{˜}{\nu }}_{ji}\left(t,T\right)∼N\left(0,\sigma_{\nu }^{2}\left(t\right)\right)\\ \mathrm{cov}\left\{{\underset{˜}{\nu }}_{ji}\left(t_{1},T\right),{\underset{˜}{\nu }}_{ji}\left(t_{2},T\right)\right\}=\{\begin{array}{c} \sigma_{\nu }^{2}\left(t\right);\;t_{1}=t_{2}=t\\ 0;\;\;t_{1}\neq t_{2} \end{array};\;\;\mathrm{cov}\left\{{\underset{˜}{\nu }}_{ji}\left(t,T_{1}\right),{\underset{˜}{\nu }}_{ji}\left(t,T_{2}\right)\right\}=\{\begin{array}{c} \sigma_{\nu }^{2}\left(t\right);\;T_{1}=T_{2}\\ 0;\;\;T_{1}\neq T_{2} \end{array}\\ \mathrm{cov}\left\{{\underset{˜}{\nu }}_{ji},{\underset{˜}{\nu }}_{lk}\right\}=\{\begin{array}{c} \sigma_{\nu }^{2}\left(t\right);\;i=k;j=l\\ \;0;\;\;i\neq k;j\neq l\;. \end{array} \end{array}$$

Because in the case of weak targets, the modulation effects will also be quite weak, the major challenge will be to detect the time-variable targets under noise conditions. Many methods are investigated with that goal [29,30,31,32,33,34,35,36,37]. Here, for the sake of brevity, we will only consider the most effective method, which is based on a matched filter concept. For that purpose, we pick out the data samples captured at an arbitrary time point $t_{0}$ and call the related signal x(T). Removing the DC-value $\bar{b_{ii}\left(t_{0}\right)}$, x(T) may be decomposed into an observation time-dependent modulation function χ(T) and a propagation time-dependent value $x_{0}\left(t_{0}\right)$ that determines the strength of the modulation dependent on the sample position as follows:

(19) $$\begin{array}{ll} x\left(T\right) & =x_{0}\left(t_{0}\right)\;\chi \left(T\right)+{\underset{˜}{\nu }}_{x}=b_{ii}\left(t_{0},T\right)-\bar{b_{ii}\left(t_{0}\right)}\\ & =\{\begin{array}{l} \;{\underset{˜}{\nu }}_{x}\;\;\;\;\;\;\;\;\mathrm{case}\;1\;\\ b_{ii}\left(t_{0}\right)\cdot \chi \left(T\right)+{\underset{˜}{\nu }}_{x}\;\;\;\mathrm{case}\;2;\;\chi \left(T\right)=\Delta \Lambda \left(T\right)/\Lambda_{0}\\ {\dot{b}}_{ii}\left(t_{0}\right)\cdot \chi \left(T\right)+{\underset{˜}{\nu }}_{x}\;\;\;\mathrm{case}\;3\text{:}\;\chi \left(T\right)=2\Delta r_{iq}\left(T\right)/c\\ \;{\ddot{b}}_{ii}\left(t_{0}\right)\cdot \chi^{2}\left(T\right)+{\underset{˜}{\nu }}_{x}\;\;\;\;\mathrm{case}\;4\text{:}\;\chi \left(T\right)=\sqrt{2}\Delta r_{iq}\left(T\right)/c\;. \end{array} \end{array}$$

In case 1, the selected time sample is located within a flat part of the receiving signal, so we get only noise ($x_{0}=0$). In case 2, the time sample is located close to a signal peak, and the time variance is caused from a variation of the target reflectivity. Cases 3 and 4, refer to a weakly moving target if the sampling time is placed on a signal edge (case 3; i.e., one of the blue signals in Figure 7B) or close to a signal peak (case 4; i.e., one of the red signals in Figure 7B). Case 4 is mostly out of interest and will not be considered further.

In order to suppress the noise, the deterministic part $x_{0}\left(t_{0}\right)\;\chi \left(T\right)$ in Equation (19) has to be emphasized. Assuming we know approximately the time shape of the modulation function, except for an unknown time delay $\zeta \left(T\right)\approx \chi \left(T-\tau_{0}\right)$, this is the case when the test scenario may be externally modulated by the operator or, in the case of heartrate and breathing detection, where one can assume an approximately sinusoidal modulation. From the assumed modulation function and the measurement data, one can establish a summation over the observation time period $T_{0}=N\;\Delta T$ (ΔT = repetition interval between consecutive measurements, see Figure 7; N = number of repetitions), which finally represents a cross-correlation function as follows:

(20) $$C_{x\zeta }\left(k\right)=\sum_{n=1}^{N}x\left(n\Delta T\right)\zeta \left(n\Delta T-k\Delta T\right)=\sum_{n=1}^{N}\left(x_{0}\left(t_{0}\right)\chi \left(n\Delta T\right)+{\underset{˜}{\nu }}_{x}\right)\zeta \left(n\Delta T-k\Delta T\right)\;.$$

Using Equation (18), its expected value and variance result in the following:

(21) $$\begin{array}{l} E\left\{C_{x\zeta }\left(k\right)\right\}=x_{0}\left(t_{0}\right)\sum_{n=1}^{N}\chi \left(n\Delta T\right)\zeta \left(\left(n-k\right)\Delta T\right)\\ \mathrm{var}\left\{C_{x\zeta }\left(k\right)\right\}=\sigma_{v}^{2}\left(t_{0}\right)\sum_{n=1}^{N}\zeta^{2}\left(n\Delta T\right)=N\sigma_{v}^{2}\left(t_{0}\right)\zeta_{rms}^{2}\;. \end{array}$$

In the case of a perfect match between modulation and reference function $\zeta \left(T\right)=\chi \left(T-\tau_{0}\right)$, the signal-to-noise ratio at a given time sample $t_{0}$ yields the following:

(22) $$SNR\left(t_{0}\right)=\frac{\max\left(E^{2}\left\{C_{x\zeta }\left(k\right)\right\}\right)}{\mathrm{var}\left\{C_{x\zeta }\left(k\right)\right\}}=\frac{{\left(x_{0}\left(t_{0}\right)N\zeta_{rms}^{2}\right)}^{2}}{N\sigma_{\nu }^{2}\left(t_{0}\right)\zeta_{rms}^{2}}=N\zeta_{rms}^{2}\frac{x_{0}^{2}\left(t_{0}\right)}{\sigma_{v}^{2}\left(t_{0}\right)}.$$

As seen from Equation (22), the detection performance can be arbitrarily increased by extending the integration time $T_{0}$ (i.e., increasing N). However, for vital data detection, the modulation is not stable over time. Hence, $T_{0}$ should not be selected too long in order to avoid de-correlation [38].

In the simplest case of sinusoidal modulation, the correlation function (20) is implemented by a Fourier-transform in observation time direction (ϕ—“observation time” frequency) as follows:

(23) $$\left|{\underline{B}}_{ii}\left(t,\phi \right)\right|=\int_{T_{0}}b_{ii}\left(t,T\right)e^{-j2\pi \phi T}dT.$$

**Figure 8:**
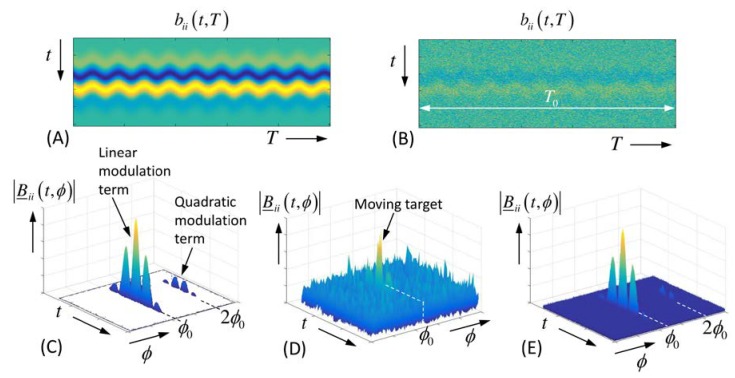
Noise-affected radar data of a sinusoidal moving point target (frequency $\phi_{0}$) according to Figure 7. (**A**) Radargram of noise-free data. (**B**) Radargram of noise-affected data. (**C**) Spectrum of noise-free radar data. Note also the quadratic term. The larger the range variation, the stronger the higher harmonics will be [39]. (**D**) Spectrum of noise-affected data integrated over $T_{0}$. (**E**) Spectrum for integration time $100T_{0}$.

Figure 8 depicts an example. Obviously it is hardly possible to identify the moving target in the original radar data if it is too noisy. However, already the correlation over the short duration $T_{0}$ makes the target visible, and further enlargement of the integration time increasingly suppresses the noise.

In many cases, however, the target modulation is not predictable in advance. Hence, the question arise from where to take the reference modulation in Equation (20). One possible option is to illuminate the target from a second antenna at a slightly different position. The resulting radar data is $b_{jj}\left(t,T\right)$. According to Equation (19), we again pick out data samples captured at $t_{1}$ and call the related signal y(T) as follows:

(24) $$y\left(T\right)=b_{jj}\left(t_{1},T\right)-\bar{b_{jj}\left(t_{1}\right)}=y_{0}\left(t_{1}\right)\;\chi \left(T\right)+{\underset{˜}{\nu }}_{y}\;.$$

Because the antennas observe the same object, the modulation function χ(t) is identical in x(t) and y(t). Taking the cross energy from both signals, we get the following:

(25) $$\begin{array}{ccc} \;E_{yx}\left(t_{0},t_{1}\right) & = & \sum_{n=1}^{N}y\left(n\Delta T\right)\;x\left(n\Delta T\right)=\sum_{n=1}^{N}\left(y_{0}\left(t_{1}\right)\chi \left(n\Delta T\right)+{\underset{˜}{\nu }}_{y}\right)\;\left(x_{0}\left(t_{0}\right)\;\chi \left(n\Delta T\right)+{\underset{˜}{\nu }}_{x}\right)\\ E\left\{E_{yx}\left(t_{0},t_{1}\right)\right\} & = & x_{0}\left(t_{0}\right)\;y_{0}\left(t_{1}\right)\sum_{n=1}^{N}\chi^{2}\left(n\Delta T\right)=Nx_{0}\left(t_{0}\right)\;y_{0}\left(t_{1}\right)\chi_{rms}^{2}\\ \mathrm{var}\left\{E_{yx}\left(t_{0},t_{1}\right)\right\} & = & \sum_{n=1}^{N}\mathrm{var}\left\{y_{0}\chi {\underset{˜}{\nu }}_{x}+x_{0}\;\chi {\underset{˜}{\nu }}_{y}+{\underset{˜}{\nu }}_{y}{\underset{˜}{\nu }}_{x}\right\}\\ & = & N\left(\left[y_{0}^{2}\left(t_{1}\right)\sigma_{x}^{2}\left(t_{0}\right)+x_{0}^{2}\left(t_{0}\right)\sigma_{y}^{2}\left(t_{1}\right)\right]\chi_{rms}^{2}+\sigma_{x}^{2}\left(t_{0}\right)\sigma_{y}^{2}\left(t_{1}\right)\right)\;. \end{array}$$

Assuming the target modulation at both signals is largest at the sampling points $t_{0},\;t_{1}$ and both receivers have identical noise behavior, we can state that $y_{0}\left(t_{1}\right)\approx x_{0}\left(t_{0}\right)=x_{0}$ and $\sigma_{x}^{2}\left(t_{0}\right)\approx \sigma_{y}^{2}\left(t_{1}\right)=\sigma^{2}$, so the signal-to-noise ratio becomes the following:

(26) $$SNR\left(t_{0},t_{1}\right)=\frac{E^{2}\left\{E_{yx}\left(t_{0},t_{1}\right)\right\}}{\mathrm{var}\left\{E_{yx}\left(t_{0},t_{1}\right)\right\}}=\frac{{\left(Nx_{0}^{2}\chi_{rms}^{2}\right)}^{2}}{N\left(2x_{0}^{2}\chi_{rms}^{2}\sigma^{2}+\sigma^{4}\right)}\approx \frac{1}{2}N\chi_{rms}^{2}\frac{x_{0}^{2}}{\sigma^{2}},$$

which gives a result of SNR only twice as poor as in the ideal case for Equation (22).

**Figure 9:**
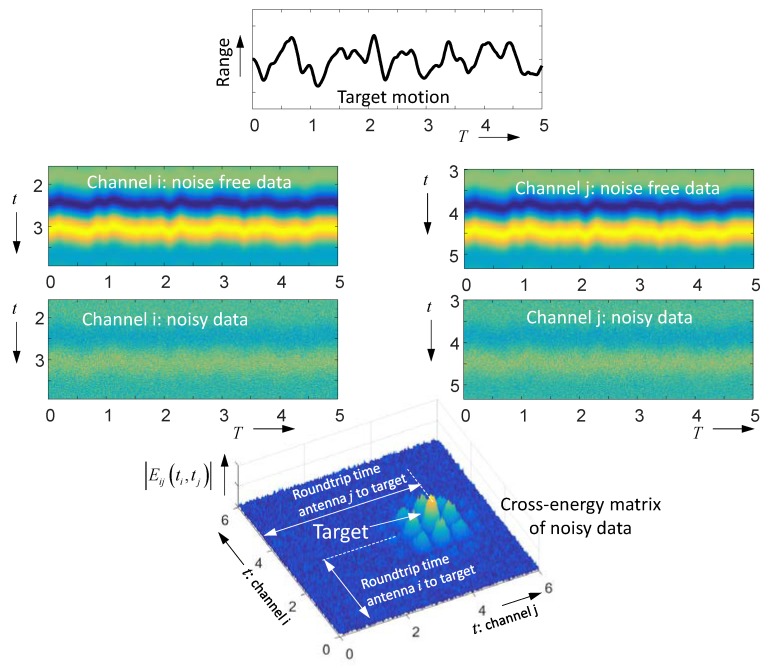
Cross-energy matrix for two independent measurements of a single moving target.

Because the optimum sampling positions $t_{0},\;t_{1}$ are not known prior, one has to run through all possible combinations leading to the cross-energy matrix, as illustrated in Figure 9. To calculate the [M,M] cross-energy matrix $E_{ij}$, we assume that the radar data are given by two [M,N] matrices $B_{ii}$ and $B_{jj}$ (M = number of samples in propagation time;N = number of impulse responses measured during the observation interval $T_{0}$). In a first step, the DC-value of every row is removed from both matrices leading to ${\overset{⌣}{B}}_{ii}$ and ${\overset{⌣}{B}}_{jj}$. Finally, from this, the cross-energy is calculated from the following:

(27) $$E_{ij}={\overset{⌣}{B}}_{ii}\;{\left({\overset{⌣}{B}}_{jj}\right)}^{T}\;.$$

The maximum position of that matrix gives the roundtrip times from both antennas. The idea behind the cross-energy is the noise independency of the merged signals, so with increasing integration time, the noise will mutually cancel out. Such noise independency, we also observe in single antenna measurements as long as the signals are not joined with themselves (i.e., the diagonal elements of the cross-energy matrix have to be set zero).

(28) $$E_{ii}={\overset{⌣}{B}}_{ii}\;{\left({\overset{⌣}{B}}_{ii}\right)}^{T};\;E_{ii}\left(k,k\right)=0\;.$$

Singular value decomposition is also often proposed for noise reduction purposes. It will, however, not help if the signal to be detected is already buried beneath noise.

### 2.3. Time-Variant Objects in Multi-Path Environment

**Figure 10:**
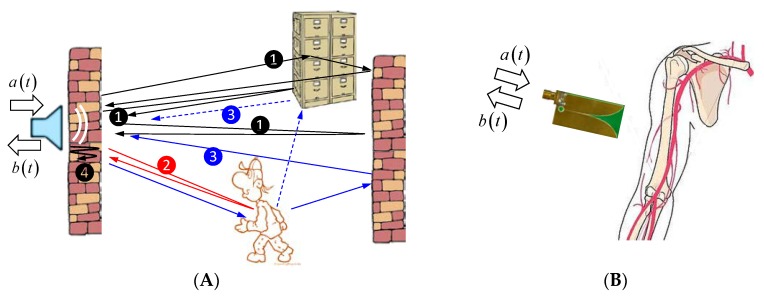
Illustration of time-variant multipath scenario. (**A**) Through-wall radar to detect humans behind walls; (**B**) Medical microwave imaging to detect artery pulsation (e.g., in the upper arm of a human).

The experimental situation is roughly illustrated in Figure 10 by two examples. One of them is with mostly free space propagation, real static objects (wall, furniture), and a target, which moves over distances much larger than the radar range resolution. The second example deals with minor motion detection (much smaller than the radar range resolution) and a wave propagation in a lossy environment, which is not very stable, because a person is not able to keep limbs truly motionless. 

We first restrict ourselves to a stable propagation environment, as depicted in Figure 10A. If threefold and higher order reflections are omitted, we can identify four different types of propagation paths. The first type ① refers to all paths, which only involve static objects. The second ② is linked with the object of interest, and the third ③ contains a twofold scattering, one by the target and one by a static object. The forth transmission path ④ symbolizes the transmission behavior of the wall, which affects the antenna signal (e.g., by multiple wall reflections). Merging all this together, the received signal for a single antenna arrangement may be modeled in simplified form as:

(29) $$\begin{array}{c} b\left(t,T\right)=T\left(t\right)*\Xi \left(t\right)*\left(\Lambda_{S}\left(t-\tau_{S}\right)+\Lambda_{T}\left(t-\tau_{T}\left(T\right),T\right)+\Lambda_{S}\left(t-\tau_{ST}\right)*\Lambda_{T}\left(t,T\right)\right)\\ {}*\Xi \left(t\right)*R\left(t\right)*a\left(t\right)+\underset{˜}{\nu }\left(t,T\right)\\ =b_{S}\left(t-\tau_{S}\right)+b_{T}\left(t-\tau_{T}\left(T\right),T\right)+b_{ST}\left(t-\tau_{ST}\left(T\right),T\right)+\underset{˜}{\nu }\left(t,T\right)\;. \end{array}$$

Here, we neither respect any angular dependency of the impulse response function nor the range influence (i.e., wave spreading and attenuation) of the signal magnitude or the polarization of the electric fields. Without loss in general, we only involve one static and one time-variable target. The different symbols stand for antenna impulse responses T(t), R(t), wall transmission Ξ(t), scattering behavior of the static object $\Lambda_{S}\left(t\right)$, and scattering of time-variable object $\Lambda_{T}\left(t,T\right)$. The symbol τ indicates the related path propagation time. In Equation (2), the path delay was respected by convolution with Dirac functions. Here, it is part of the arguments of the function to shorten the notation. In the bottom line of Equation (29), the different components of the transmission paths are merged into different functions. $b_{S}\left(t\right)$ symbolizes all transmission paths (including multiple reflections), which do not change in observation time. This part represents the strongest component of the measured signal. It is often orders of magnitude larger than the other components. $b_{T}\left(t,T\right)$ refers to the time-variable target, in which we are actually interested, and $b_{ST}\left(t,T\right)$ represents multipath components, which involve the time-variant target. $b_{S}\left(t\right)$ and $b_{ST}\left(t,T\right)$ are often assigned as clutter. They must be removed from the measured signal.

Before we do that, the noise term in Equation (29) must be accounted for more seriously, because this will be important for the estimation of the clutter reduction. As already mentioned in Equation (17), the randomness of the measurement is caused by amplitude noise (additive noise) $\underset{˜}{n}∼N\left(0,\sigma_{n}^{2}\right)$ and “time” noise (i.e., sampling jitter) $\Delta {\underset{˜}{\tau }}_{j}∼N\left(0,\varphi_{j}^{2}\right)$. In well-designed receiver electronics, both can be assumed to be Gaussian distributed, white, independent, and ergodic. By considering both noise terms separately, Equation (29) has to be modified to the following:

(30) $$b\left(t,T\right)=b_{S}\left(t+\Delta {\underset{˜}{\tau }}_{j}\right)+b_{T}\left(t+\Delta {\underset{˜}{\tau }}_{j},T\right)+b_{ST}\left(t+\Delta {\underset{˜}{\tau }}_{j},T\right)+\underset{˜}{n}\left(t,T\right)\;.$$

The propagation delay is omitted in the signal components for the sake of a shorter notation. Due to the ergodicity of the additive noise, the randomness of the sampling procedure does not influence its statistical properties, where the jitter $\Delta {\underset{˜}{\tau }}_{j}$ is omitted in the noise term $\underset{˜}{n}\left(t,T\right)$.

In order to suppress the static paths, one needs to know $b_{S}\left(t\right)$. It can either be determined from measurements where the target is still absent or it is estimated by averaging over the captured data. In both cases, one performs an integration in observation time in order to reduce the noise influence. So, we can approximately write the following:

(31) $$\bar{b_{S}\left(t\right)}\approx \frac{1}{T_{0}}\int_{T_{0}}b\left(t,T\right)dT\approx b_{S}\left(t\right)*p_{\Delta \tau }\left(t\right).$$

Equation (31) assumes that the variable target parts are canceled out due to a sufficiently long integration. Furthermore, it shows that the time shape of the static signal is slightly modified by “a low pass filter” whose IRF is given by the PDF $p_{\Delta \tau }\left(t\right)$ of the jitter. However, this becomes only remarkable if the standard deviation $\varphi_{j}$ of the jitter is on the order of the rise time of the signals. The additive noise in Equation (31) is omitted, because it is largely suppressed by the averaging. In practical implementation, the integration in Equation (31) may be performed over the whole captured data set or even by slighting averaging or low pass filtering the observation time. The signal $\bar{b_{S}\left(t\right)}$ is often assigned as background.

By subtracting $\bar{b_{S}\left(t\right)}$ from the measured data, the result after some manipulation is as follows:

(32) $$\begin{array}{cc} c\left(t,T\right) & =b\left(t,T\right)-\bar{b_{S}\left(t\right)}\\ & \approx b_{S}\left(t\right)\;*\left(\delta \left(t\right)-p_{\Delta \tau }\left(t\right)\right)+{\dot{b}}_{S}\left(t\right)\Delta {\underset{˜}{\tau }}_{j}+b_{T}\left(t,T\right)+{\dot{b}}_{T}\left(t,T\right)\Delta {\underset{˜}{\tau }}_{j}+b_{ST}\left(t,T\right)+{\dot{b}}_{ST}\left(t,T\right)\Delta {\underset{˜}{\tau }}_{j}+\underset{˜}{n}\\ & \approx {\dot{b}}_{S}\left(t\right)\;\varphi_{j}+{\dot{b}}_{S}\left(t\right)\Delta {\underset{˜}{\tau }}_{j}+b_{T}\left(t,T\right)+b_{ST}\left(t,T\right)+\underset{˜}{n}\;. \end{array}$$

The second line in Equation (32) comes from a Taylor series expansion of Equation (30) and the assumption of a not significantly large jitter, so higher terms of the series may be neglected. Further on, the third line ignores the jitter-affected time-variant signals, because they are of very low magnitude, and $p_{\Delta \tau }\left(t\right)$ is approximated by the first element of its Taylor series.

**Figure 11:**
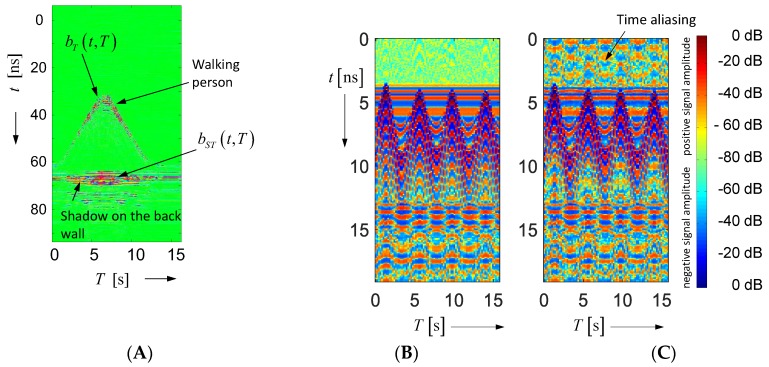
Illustration of multipath influence onto radar data: (**A**) Radar data of a moving person after background removal. The horizontal signal trace represents the third path illustrated in Figure 10: antenna to person, forward scattering of person to the back wall and back to the antenna. (**B**) Radar data after background removal gained from moved sheet metal. The experiment was done in an ordinary laboratory space with a 12th order M-sequence device (total length of pulse response 307 ns). (**C**) Measurement done with 8th order M-sequence device showing a too short unambiguity range (length of pulse response 19 ns; see also Section 3.3).

As we can observe from Equation (32), we get after background removal the wanted signal $b_{T}\left(t,T\right)$, but it is still bothered by other components. One of them is the multipath component $b_{ST}\left(t,T\right)$. It is illustrated in Figure 11. Figure 11A is based on the same scenario as already discussed in Figure 6, but now it refers to the completely captured data set. Because the person was walking in a room, it created a shadow on the wall opposite to the antenna, which may have the same strength as the wanted signal. However, the signal from the shadow has a larger propagation time compared to the direct target reflection. This gives us the opportunity to single out the signal from the shadow and the direct target reflection. This will work better with a larger bandwidth of radar, because due to the better range resolution, targets close to the wall may be separated from their shadows more easily.

Then, we still have the additive noise $\underset{˜}{n}$ in Equation (32) affecting the detection performance. This noise has been treated in many previous papers and will hence not be considered here in detail. A more serious effect comes from the jitter, which leads to the bias term ${\dot{b}}_{S}\left(t\right)\;\varphi_{j}$ and the random term ${\dot{b}}_{S}\left(t\right)\Delta {\underset{˜}{\tau }}_{j}$. The bias term is independent of the observation time and thus will be less critical for the target detection (note that $\varphi_{i}$ is independent of t and T). The opposite is valid for the random term. $\Delta {\underset{˜}{\tau }}_{j}$ is random in t and T. Furthermore, its random effect on c(t,T) is weighted by the first derivative ${\dot{b}}_{S}\left(t\right)$ of the very strong signal scattered from the static objects. Under strong multipath conditions, these signals are typically spread over the whole radar range. Figure 11B gives an example of signal spreading even under simple conditions. To get a better impression of the process of dying out, the data are logarithmically scaled by keeping the signal sign. The scaling function is given by $b_{\log}=\mathrm{sign}\left(b\right)\cdot \left(\max\left[20\mathrm{lg}\left(b/b_{\max}\right),-D\right]+D\right)$. D [dB] is the dynamic range over which the data are depicted.

The strength of the background is often 2–3 orders of magnitude larger than the target reflections and with increasing bandwidth of the radar, the signal derivation leads to an additional emphasis of their influence. Hence, jitter may seriously affect the detection performance of a time-variable target under strong multipath conditions (especially if it is at the same range as a strong target—compare Figure 10—person, and file cabinet). UWB radar experiments for motion detection under (nearly) free space conditions are therefore less trustworthy.

So far, we have assumed that the clutter objects are static. In a situation as depicted in Figure 10B, this cannot be strictly presumed, because a living organism can never suppress completely the motion of its limbs. Hence, by referring to the signal model in Equation (30), we also have to take into account a minor time variance of the signal component $b_{s}\left(t\right)\to b_{s}\left(t,T\right)$. Under the condition that all motion effects are small (i.e., time-delay modulations are smaller than the rise time of the sounding signal), we can follow the approach introduced with respect to Equation (19). That is, we pick out data samples at different propagation timepoints $t_{k}$ and observe their magnitude dependent on the observation time $b\left(t_{k},T\right)$ or $c\left(t_{k},T\right)$. The modulation of the majority of data samples will follow the global motion of the scenario under testing conditions (e.g., the arm), and only few data samples will also contain a modulation caused by the target of interest. The goal is to separate both modulations and to extract the target.

Under the assumption that both modulations are independent or orthogonal, respectively, this can be done by principal component analysis (PCA), which exploits singular value decomposition (SVD) [40].

**Figure 12:**
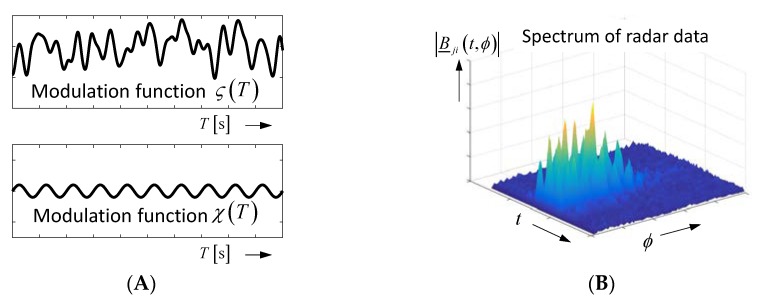

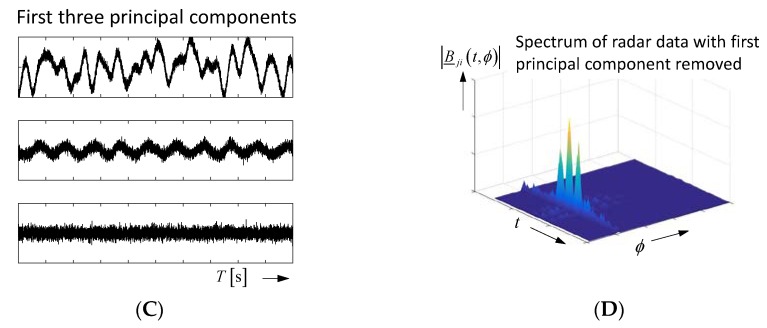
Separation of time-variable clutter by principal component analysis (PCA). (**A**) Modulation functions of the radar signal. (**B**) Spectral representation of radar data (see also Figure 8). (**C**) The first three principal components of the radargram. (**D**) Spectrum after removing the first principal component.

Figure 12 gives an example. Assuming, we want to measure the artery motion in the arm. It is approximated in our example by the sinusoidal modulation χ(T) (Figure 12A). Due to the vital motion of the body, we get an additional, irregular modulation ζ(T), which is even stronger than the signal of interest. Both modulations overlap in the radar signal, but they are connected with slightly different roundtrip times. These two signals affect the radar signal in the same way, as depicted in Figure 7. Because we are interested in a periodic motion, FFT (Fast Fourier Transform) is performed in observation time with the hope of finding the wanted signal (Figure 12B). However, we do not succeed, because the perturbing modulation ζ(T) suppresses the modulation χ(T) of interest. Exploiting PCA on the radargram leads to two separable principal components (Figure 12C). Because the second principal component meets best our expectation about the wanted signal, the related signal parts are extracted from the radar signal. Now, the spectrum shows the wanted sinusoidal modulation signal (Figure 12D).

## 3. Device Requirements for Differential Imaging

This section will summarize the requirements for the most important parameters of UWB devices for differential imaging. Basically, there are several approaches possible to implement UWB devices [1]. They differ mainly by the test signal under use, such as stepped sinewave, sub-nanosecond pulse, binary pseudo random code, multi-sine, and random noise. Seen from the perspective of implementation cost, bandwidth, measurement speed, and multi-channel capability, however, only two approaches will remain the focus of interest. These are the short pulse and pseudo random noise excitation. The last one mostly applies M-sequences, but basically, it is not restricted to that type of pseudo random code. In what follows, we only include these two principles in our discussion.

### 3.1. Pulse and M-Sequence Radar

**Figure 13:**
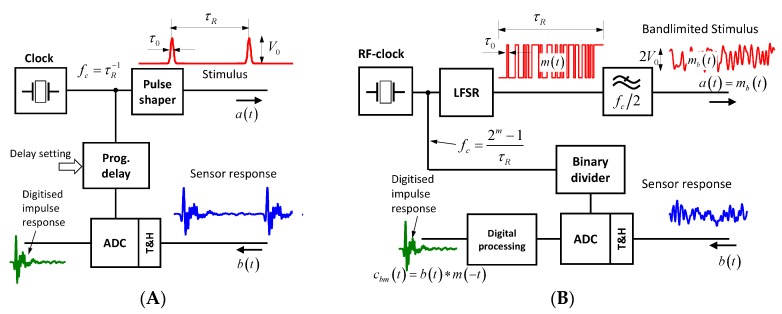
Block schematic of pulse radar (**A**) and M-sequence radar (**B**). Both principles are working in the baseband. For extensions to bandpass principles see [1,41]. Note that the actual sounding signal of an M-sequence radar is the bandlimited M-sequence $m_{b}\left(t\right)$.

The basic structure of pulse and M-sequence are depicted in Figure 13. The pulse radar (Figure 13A) is based on a pulse shaper, which launches periodically sub-nanosecond pulses triggered by a clock generator of repetition rate $\tau_{R}^{-1}$. The data capturing is organized via subsampling in order to reduce the sampling rate of the ADC (Analog to digital converter). In the shown example, sequential sampling is used, by which one data sample per launched pulse is captured. By a programmable delay line, the sampling point is moved over the time interval of interest $\tau_{ROI}\leq \tau_{R}$. The step size of the delay variation determines the equivalent sampling rate $\Delta \tau =f_{eq}^{-1}$, which has to meet the Nyquist sampling criteria for the sounding signal. This is not required from the actual sampling rate. For modifications of the sampling principle (e.g., interleaved sampling) see [1,41]. The peak power of the transmitted signal $\hat{P}$, its average power P, and the achievable bandwidth $\underset{¨}{B}$ (note $\underset{¨}{B}$ is a two-sided bandwidth) are as follows:

(33) $$\hat{P}∼V_{0}^{2};\;\;P∼\frac{\tau_{0}}{\tau_{R}}V_{0}^{2};\;\;\underset{¨}{B}\;\tau_{0}\approx 1.$$

In the case of the M-sequence radar the sounding signal is generated by a fast linear feedback shift register (LFSR) of length m, which is pushed by a microwave source of frequency $f_{c}$. It provides $N_{m}=2^{m}-1$ chips per period. For data gathering, an interleaved subsampling approach is used. Due to spectrum limitations below $f_{c}/2$, the equivalent sampling rate is preferentially selected to $f_{eq}=f_{c}$ so that a binary divider can simply control the sampling [1]. Transmitter power and double-sided bandwidth are as follows:

(34) $$\hat{P}\approx P∼V_{0}^{2};\;\;\underset{¨}{B}=f_{c}=\frac{1}{\tau_{0}}.$$

However, the received signal b(t) is mostly worthless as it leaves a chaotic impression. Therefore, one has to take its circular cross-correlation $c_{bm}\left(t\right)$ with the M-sequence code m(t) for further data processing [1] in order to get the wanted impulse response function as follows:

(35) $$c_{bm}\left(t\right)=\int_{0}^{\tau_{R}}b\left(\xi \right)m_{c}\left(\xi +t\right)d\xi \;.$$

### 3.2. Bandwidth, Measurement Rate, and Antenna Array

The bandwidth of the sounding signal determines the range resolution performance of the radar. Hence, it should be as large as possible. The bandwidth is fixed by the width of the sounding pulse or the chip width of the M-sequence, respectively. The upper-band limits are, however, often restricted by the frequency-dependent propagation losses of the test scenario. Because these losses grow with the increasing target distance, the range resolution will worsen the deeper a target is located in a lossy material.

The measurement rate $\phi_{R}$ has to respect the Nyquist sampling criteria of the temporal target variation. For heart rate monitoring, for example, at least several tens of impulse response functions per second have to be collected if one is also interested in the higher harmonics of the heart motion.

In the UWB case, λ/2-antenna spacing is not an issue, so often a low number of antenna positions is already sufficient for localization purposes in a scenario with a low number of targets. The antennas must be kept in stable positions, because even minor movements cause signal modulation, which represses the target modulation. In order to permit measurements in highly time-varying scenarios, parallel operation of all receiver channels is required. To illuminate the scenario from different aspect angles, several transmitting antennas need to be involved. Because orthogonal UWB signals are usually not available or difficult to generate, the antennas are not allowed to transmit in parallel. Consequently, the different transmitters have to be activated sequentially. This lowers the measurement rate wherefore the number of transmitters should be limited to the minimum, because the Nyquist sampling criteria for the temporal target variation applies to the whole cycle of data collection.

### 3.3. Unambiguity Range and Data Throughput

The sounding signals, pulse, or M-sequence, are periodically repeated with the interval $\tau_{R}$. All targets whose distances to the antenna are shorter than

(36) $$R_{0}=\frac{\tau_{R}c}{2}$$

can be unambiguously arranged to their correct range by radar measurements. In UWB imaging scenarios, one is often only interested in a small observation area or volume, so the distances of interest are quite short. In breast cancer imaging, for example, a range of about 10 cm would be already sufficient. This may lead to the suspicion that an unambiguity range of a bit larger than 10 cm could be sufficient. But, that does not take into account the unwanted transmission paths at the antenna-feeding cable, multipath propagation, or antenna back radiation into the surrounding space. Signals, which are subjected to a delay larger than $\tau_{R}$, are folded in the captured principal signal segment and appear as “ghosts”, which are not distinguishable from signals with the correct delay. As long as the ghosts do not vary in observation time, they are not critical in differential imaging, because they will be eliminated by the background removal in Equation (32). However, because the object motions in the surrounding of an imaging experiment are mostly not under the control of the operator, they will affect sensitive measurements if they are not able to die off within the time window $\tau_{R}$. Figure 11C illustrates an example of a too short time interval $\tau_{R}$, which did not allow the waves to die out.

The total net data throughput H [bits/s] of the receivers of a differential imaging system results from the following:

(37) $$H=N_{B}N_{S}K\;L\;\phi_{R}$$

where $N_{B}$ is the word length of a data sample, $N_{S}$ is the number of data samples per response functions, K, L are the number of transmitter and receiver channels, respectively, and $\phi_{R}$ is the measurement rate. In order to meet the Nyquist rate of IRF acquisition, the minimum number of data samples $N_{s}$ to be collected is as follows ($\underset{¨}{B}$ is the double-sided bandwidth):

(38) $$N_{s}\approx \{\begin{array}{c} \tau_{ROI}\underset{¨}{B}\;\;\mathrm{pulse}\;\mathrm{radar}\;\;\\ \;\tau_{R}f_{c}=N_{m}\;\;\;M-\mathrm{sequence}\;\mathrm{radar}\;. \end{array}$$

While an M-sequence radar has to collect the data over the full period $\tau_{R}$ of the sounding signal, a pulse radar can be limited to a time window of interests $\tau_{ROI}$. Note that Equation (38) represents an absolute minimum. In the case of pulse radar, one records usually many more samples in order to get a quasi-continuous impression of the signal. If fast ADCs are applied, one is able to collect more samples as required within the time interval $\phi_{R}^{-1}$, which should be used to improve the noise behavior by synchronous averaging.

Finally, it should be noted that in case of a large unambiguity range (i.e., large $\tau_{R}$), the average power of a pulse radar will drastically decrease—compare with Equation (33)—while for an M-sequence radar, such an effect will not be observed.

### 3.4. Random Effects

As already mentioned above, UWB devices are subjected to two types of randomness. These are the uncertainties of voltage capturing due to thermal noise and quantization effects symbolized by $\underset{˜}{n}$ and the uncertainties (jitter) of the sampling time $\Delta {\underset{˜}{\tau }}_{j}$.

Assuming a frequency-independent noise power spectral density $\Phi_{n}$, the power of the additive noise $P_{n}=\mathrm{var}\left\{\underset{˜}{n}\right\}=\sigma_{n}^{2}\approx \Phi_{n}\underset{¨}{B}$ will rise with the bandwidth $\underset{¨}{B}$. The measures to combat against the impact of this noise are to increase the average power of the sounding signal and to emphasize the deterministic measurement effects against the noise by an appropriate integration over a long observation time (e.g., synchronous averaging; matched filtering—compare Equation (20) and Figure 8 and Figure 9).

**Figure 14:**
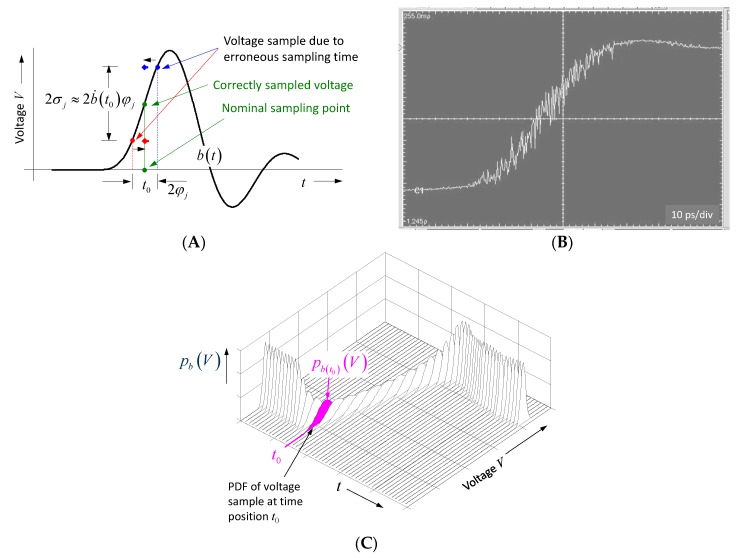
Impact of sampling jitter on data capturing. (**A**) Illustration of the effect at a single sampling point $t_{0}$. (**B**) Measurement example. (**C**) Probability density functions of a rising signal edge.

The impact of sampling jitter is illustrated in Figure 14. Sampling jitter causes data capturing at incorrect and unknown time instances. Because the captured voltage has to be assigned to the pre-defined sampling time $t_{0}$, the timing uncertainty transforms into amplitude noise dependent on the signal slope. The integral effect of the jitter on the signal quality may be expressed by the signal-to-noise ratio relating the average signal power P to the jitter induced power $P_{j}$ as follows:

(39) $$SNR_{j}=\frac{P}{P_{j}}=\frac{\frac{1}{\tau_{R}}\int_{\tau_{R}}b^{2}\left(t\right)dt}{\frac{\varphi_{j}^{2}}{\tau_{R}}\int_{\tau_{R}}{\dot{b}}^{2}\left(t\right)dt}=\frac{\int_{-\infty }^{\infty }{\left|\underline{B}\left(f\right)\right|}^{2}df}{4\pi^{2}\varphi_{j}^{2}\int_{-\infty }^{\infty }f^{2}{\left|\underline{B}\left(f\right)\right|}^{2}df}\approx \frac{3}{\pi^{2}\varphi_{j}^{2}{\underset{¨}{B}}^{2}}.$$

${\left|\underline{B}\left(f\right)\right|}^{2}$ represents the power spectrum of the sounding signal. Applying Parseval’s theorem and the differentiation rule of Fourier transform and assuming for the sake of simplicity a constant spectrum of the sounding signal within the band limits $-\underset{¨}{B}/2\cdots \underset{¨}{B}/2$, one finds that the jitter effect is independent of the actual time shape and the power of the sounding signal. This result is confirmed by simulations and measurements in [42].

However, this approach obstructs the view of the actual situation. Jitter-induced noise is non-ergodic. Therefore, its actual dependency on time should be considered for better understanding of its impact. As is obvious from Figure 14, jitter develops its effect mainly on signal edges. Hence, in the case of pulse radar, the whole jitter power is concentrated at the edges of strong signal parts.

It remains the question how the output signal $c_{bm}\left(t\right)$ of an M-sequence radar (see Equation (35)) is affected by jitter and noise:

(40) $$\begin{array}{ccc} c_{bm}\left(t\right) & = & \frac{1}{\tau_{R}}\int_{0}^{\tau_{R}}\left(b_{0}\left(\xi \right)+{\dot{b}}_{0}\left(\xi \right)\Delta {\underset{˜}{\tau }}_{j}+\underset{˜}{n}\right)m_{c}\left(\xi +t\right)d\xi \\ E\left\{c_{bm}\left(t\right)\right\} & = & \frac{1}{\tau_{R}}\int_{0}^{\tau_{R}}b_{0}\left(\xi \right)m_{c}\left(\xi +t\right)d\xi \\ \mathrm{var}\left\{c_{bm}\left(t\right)\right\} & = & \frac{1}{N_{m}}\left(\sigma_{n}^{2}+\bar{{\dot{b}}_{0}^{2}}\;\varphi_{j}^{2}\right);\;\mathrm{with}\;\bar{{\dot{b}}_{0}^{2}}=\frac{1}{\tau_{R}}\int_{0}^{\tau_{R}}{\dot{b}}_{0}^{2}\left(t\right)dt\;\mathrm{and}\;\sin\mathrm{ce}\;\int_{0}^{\tau_{R}}m^{2}\left(t\right)dt=1\;. \end{array}$$

As we can observe from Equation (40), the expected value is a bias-free estimation of the wanted correlation function, and the total noise depends on the strength of the received signal due to the jitter. Unlike the pulse radar, the noise level is not focused on signal edges but is evenly distributed throughout the signal (i.e., it is converted into additive noise, which depends on the signal level and bandwidth (due to differentiation)). Additionally, the noise level decays with increasing length $N_{m}$ of the M-sequence. The maximum noise level appears if there is a direct connection between the receiver and transmitter (i.e., $b_{0}\left(t\right)=m_{b}\left(t\right)$). Assuming again constant spectral power within the stimulation band $-\underset{¨}{B}/2\cdots \underset{¨}{B}/2$ or $-f_{c}/2\cdots f_{c}/2$, respectively, we find the following:

(41) $$\begin{array}{cc} {\mathrm{var}\left\{c_{bm}\left(t\right)\right\}|}_{\max} & =\frac{1}{N_{m}}\left(\sigma_{n}^{2}+\bar{{\dot{m}}_{b}^{2}}\;\varphi_{j}^{2}\right)=\frac{1}{N_{m}}\left(\sigma_{n}^{2}+\;\varphi_{j}^{2}\int {\left|j2\pi fM_{b}\left(f\right)\right|}^{2}df\right)\\ & \approx \frac{1}{N_{m}}\left(\sigma_{n}^{2}+\;\frac{1}{3}{\left(\pi V_{0}f_{c}\varphi_{j}\right)}^{2}\right)\;. \end{array}$$

In order to stay in the digital domain, we characterize the overall sensitivity of the receiver (i.e., ADC resolution and track-and-hold noise) by its effective number of bits $ENOB=\log_{2}\left(2V/\sigma_{n}\right)$ (2 V—ADC full scale range) so that we get from Equation (41) equality between quantization/thermal noise and jitter induced noise under the following condition:

(42) $$\varphi_{j,0}\approx \frac{\sqrt{3}\;}{\pi \;f_{c}2^{ENOB-1}}\;.$$

**Figure 15:**
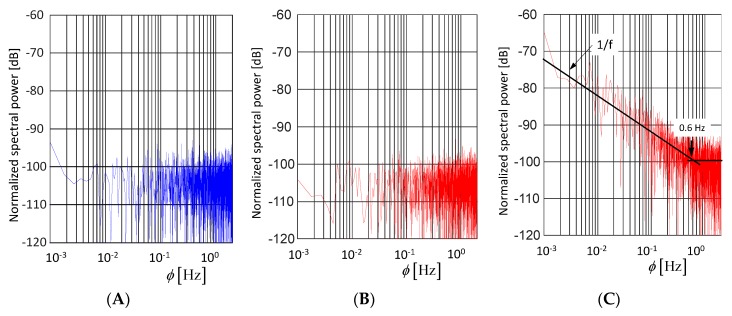
Spectral noise power of 12th order M-sequence device m: explore. (**A**) receiver input open. (**B**) maximum input signal; sample taken from flat signal part. (**C**) maximum input signal, sample taken from steepest signal part.

This is an important design rule for M-sequence devices in order to balance between resolution, technical effort, and data rate. Assuming an operational frequency band 0⋯5 GHz ($f_{c}=10\;\mathrm{GHz}$) and ENOB=10 bit, the standard deviation of the jitter should not exceed $\varphi_{j}\leq 100\;\mathrm{fs}$. This is a challenging task. Therefore, the timing concept of the M-sequence radar is specifically designed for low jitter generation (see [21]). The time reference is provided by a low-phase-noise single-tone microwave source. Other timing-critical components, such as LFSR, binary divider, and track-and-hold circuit are monolithically integrated in a high frequency SiGe-technology providing low-noise signals of steep trigger edges. Figure 15 illustrates the noise behavior of a commercial M-sequence device [43]. Figure 15A shows the pure additive noise $\underset{˜}{n}$, because no signal was fed into the receiver (blue spectrum). Figure 15B,C are showing the spectrum of the observation time noise for the maximum input signal (red spectra). Figure 15B refers to a voltage sample placed at a horizontal signal part, and Figure 15C depicts the fluctuations at the steepest signal edge. The noise level in Figure 15A,B are nearly identical. Hence, from Equation (42), the sampling jitter of the device is sufficiently small, so it does not degrade the noise performance of the device. In Figure 15C, the noise level for frequencies above ϕ≥0.6 Hz is about the same as in the two previous cases, which also confirms Equations (41) and (42). For frequencies below ϕ<0.6 Hz, the noise increases inversely with frequency. The actual reason for this behavior has still to be investigated. Because the measurement was done in a non-temperature-controlled room, one reason could be minor temperature variations, which affect the propagation time of the cable connecting transmitter and receiver. Thanks to the noise suppression of correlation (see Equation (40)), the remaining temporal fluctuation of an M-sequence device is typically in the range of few femtoseconds. Temperature fluctuations in the range of only one-hundredth of a degree lead already to runtime changes on RF-cables that exceed the measurement uncertainty of the device.

**Figure 16:**
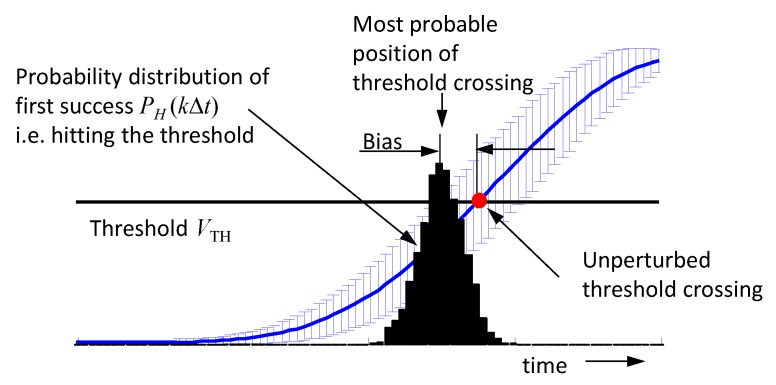
Threshold crossing of a noisy signal edge.

Finally, it should be pointed to an effect that leads to an erroneous roundtrip time measurement due to noise [44]. Assuming the roundtrip time is determined by the first crossing of a threshold. The situation is illustrated in Figure 16. The rising edge of a signal is affected by random noise, whose standard deviation is indicated by error bars. A related presentation is depicted in Figure 14C showing the PDFs $p_{b\left(i\Delta t\right)}\left(V\right)$ of the voltage samples collected at different sampling points iΔt.

Hence, the probability that a voltage sample captured at time position iΔt hits the threshold is as follows:

(43) $$P\left(i\Delta t\right)=\int_{V_{TH}}^{\infty }p_{b\left(i\Delta t\right)}\left(V\right)\;dV\;.$$

Further, the probability that the threshold is passed the first time at sample position kΔt is (probability of first success) as follows:

(44) $$P_{H}\left(k\Delta t\right)=P\left(k\Delta t\right)\prod_{i=0}^{k-1}\left(1-P\left(i\Delta t\right)\right);\;k>i\;.$$

Such a probability distribution is illustrated in Figure 16, indicating that the most probable time point for threshold crossing is located before the correct value. In order to minimize this bias error, the noise at the signal edge should be weak and the density of the sampling points should not be much larger than required by the Nyquist sampling criteria. However, the determination of the correct position of the threshold crossing then needs a suitable interpolation between the voltage samples laying on either side of the threshold.

## 4. Demonstration Examples

For illustration of the differential imaging approach, three examples shall be shown. A few further examples can be found in [45,46,47,48,49,50,51,52]. First, we consider breast cancer imaging via modulation of targeted nanoparticle. The other two examples are restricted mainly to the detection of moving objects, because this is the key issue in differential imaging.

A major problem in microwave breast cancer detection is the low contrast of the malignant tissue and the strong clutter caused from the skin and glandular tissue. In order to circumvent such clutter, it is proposed to target the malignant tissue with nanoparticles [53,54] and to observe the difference before and after the injection of such particles. Because their accumulation in the breast needs longer time, the patient may not be able to stay in the measurement position. This degrades the reproducibility of the repetition measurement [55].

**Figure 17:**
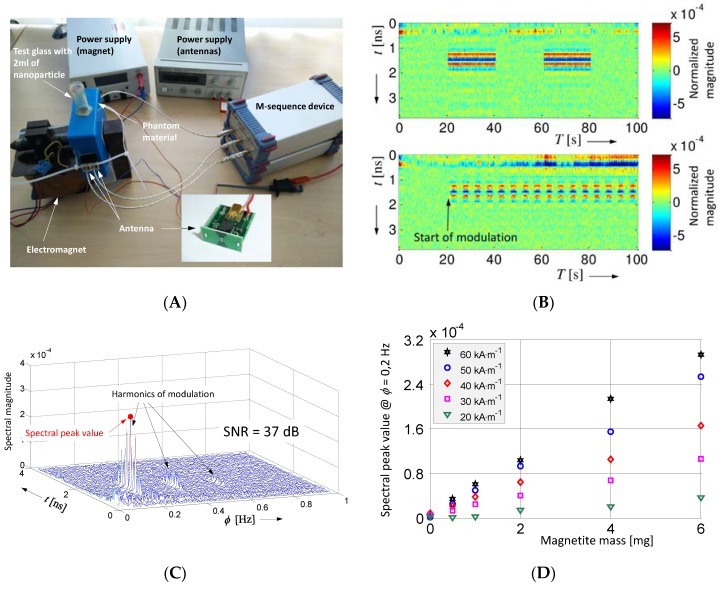
Modulation of microwave scattering of nanoparticles by an external magnetic field. (**A**) Test-setup. (**B**) Radargram for on–off keying (above) and sinusoidal H-field modulation (below). (**C**) “Observation time” spectrum for sinusoidal field modulation. (**D**) Strength of signal variation (backscattering) as a function of nanoparticle mass and magnetic field strength.

Another approach is based on nanoparticles, which can be modulated by an external magnetic field [56,57,58,59,60]. In that case, the measurements are done after accumulation of nanoparticles in the malignant tissue of the breast so that the patient may keep in position the whole time needed for the data collection. Figure 17 illustrates the basic concept and some results [57,59]. The test setup is shown in Figure 17A. A test glass is filled with 2 mL of a liquid enriched with a certain amount of magnetite nanoparticles. This glass is placed in a block of phantom material mimicking the breast tissue. Both are finally arranged between the poles of an electromagnet and the microwave backscattering, and transmission is measured with an M-sequence device. For the shown example, the path length in both cases is about 5 cm. For the radar measurements, small active antennas are applied (see inset of Figure 17A). Figure 17B shows radargram examples of the backscattered data after static background removal for on–off keying and sinusoidal modulation, respectively. The radargrams of the transmission measurement look similar. The variation of the target response is in the range of ±10^−4^ below the maximum signal magnitude caused by the strongest static propagation path (e.g., antenna cross talk or skin reflection). The radargram example refers to 6 mg of magnetite and a modulation strength of $H_{\max}=60\;\mathrm{kA}\cdot m^{-1}$. The related signal-to-noise ratios between peak value and noise floor are about 18 dB for the pulse modulation and 20 dB for the sinusoidal variation, respectively. It is evident that the signals will be lost in noise by reducing the number of nanoparticles. However, as discussed in Figure 8, we can provide a SNR-improvement by correlating (matched filtering) the radargram in the observation time direction with the modulation signal. This is depicted in Figure 17C for the sinusoidal modulation and matched filtering via Fourier transform over a measurement time of about 200 s. This improves the SNR-value to 37 dB. The actual sensitivity of the measurement arrangement is depicted in Figure 17D. It shows the spectral peak value as a function of the magnetite mass and modulation strength of the magnetic field. As demonstrated, the detection threshold is in the range of mg and can be further reduced by technical improvements and methods of SNR enhancement. The detectable mass relates to a number of nanoparticles, which can physiologically be accumulated in malignant tissue. It should further be noted that electronic components of the microwave UWB radar, which are placed close to the magnetic modulation field, should not be composed from ferromagnetic materials, such as iron or nickel.

**Figure 18:**
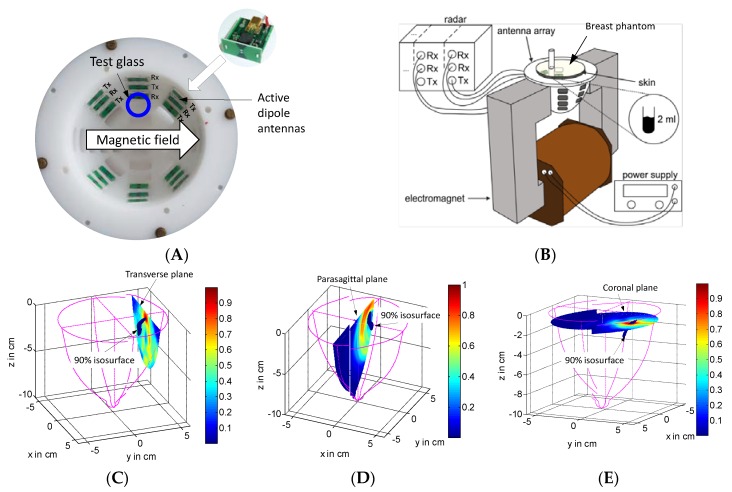
Breast cancer imaging via modulated nanoparticles. (**A**) Breast mold partially equipped with antennas. (**B**) Measurement setup. (**C**–**E**) Three-dimensional (3D)-images under different perspectives. For the sake of better illustration, the transversal and parasagittal plane do not cross the voxel of maximum intensity. But, the coronal plane includes that voxel.

The imaging concept is finally depicted in Figure 18. A breast mold is—for the sake of demonstration—partially equipped with antennas. In the shown imaging example, only the upper antennas are actually used. Four of them act as receivers, and five provide sequentially the ultra-wideband sounding field. The mold is arranged between the poles of an electromagnet and a breast phantom with a test glass of nanoparticles (WHKS 1S12, Liquids Research Limited, Bangor, UK) diluted in distilled water is placed into the mold. The liquid volume is located about 2 cm below the surface. The breast phantom consists of a 2 mm thick skin layer made by silicone mixed with Carbon Black [61,62], and the healthy tissue surrogate is an oil–gelatin mixture [63].

Figure 18C–E show the result of the three-dimensional (3D)-imaging seen from different aspect angles. The target is represented by a blue colored isosurface embracing all voxels having an intensity larger than 90% of maximum voxel intensity. The imaging algorithm is based on the delay-and-sum approach (compare Equation (4)) taking only such signal components that are modulated by the magnetic field. The imaged target creates the impression of a slightly curved rod. The different planes inserted into the breast volume nicely indicate the sidelobes of the imaging procedure, and they give an impression about the resolution. As we can observe, the resolution within the coronal plane is much better than in the other two planes. The reason is the antenna arrangement. The resolution in the coronal plane is better, because the angle in the coronal plane under which the antennas “see” the target is much larger than the related angle in the parasagittal plane.

**Figure 19:**
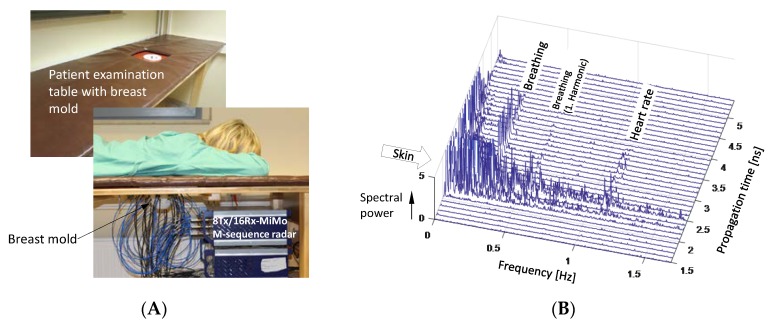
Impact of vital motions onto radar data (**A**) measurement setup (courtesy B. Faenger). (**B**) Spectral radar data. Every line represents the spectrum of an individual sampling point of the impulse response function.

In order to select an appropriate modulation frequency for the nanoparticles, the intrinsic time variance of a female breast was investigated (see Figure 19). For that purpose, a healthy female volunteer placed her left breast in the antenna mold. The volunteer person was laying in prone position, and she was not moving. The signals after background removal and Fourier transform in observation time are depicted in Figure 19B. Ideally, only noise should remain. But, not surprisingly, one finds not only the breathing rate and the heart rate, but also strong random variations at the position of the skin in the data. They may be caused by minor global body motions, which the person can never suppress if not anesthetized. Fortunately, these signals die out above 2 Hz so that a good choice for the modulation rate for the nanoparticles would fix it beyond 2 Hz.

**Figure 20:**
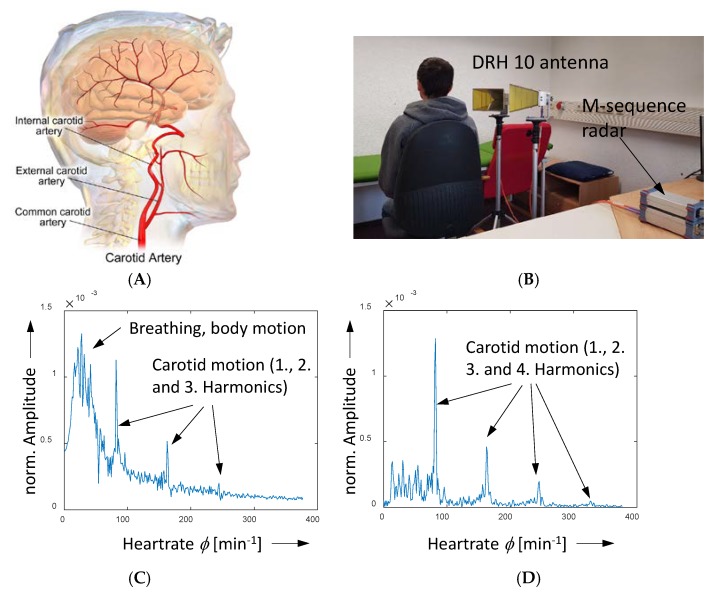
Measurement of carotid artery motion. (**A**) Anatomy of the human head (courtesy Bruce Blaus) (**B**) Measurement setup. (**C**) Spectrum of the range sample with strongest variation. (**D**) Motion spectrum after removal of unwanted neck motion.

While in the first demonstration example, the modulation rate may be appropriately selected to avoid overlap with other motions, this degree of freedom is not given in the second one. Here, the goal is to detect, for example, the motion of the carotid artery. Figure 20 depicts the measurement setup and the results. The measurements are done from a distance of about half a meter. The person is sitting in quiet position. Nevertheless, minor movements by breathing or swallow cannot be avoided by the volunteer. These are the dominant variations as it can be observed in Figure 20C. To remove their influence on the radar data, a PCA/SVD (Principal Component Analysis/Singular Value Decomposition) method may be used. The improved spectrum is shown in Figure 12D, in which the unwanted components are largely suppressed.

**Figure 21:**
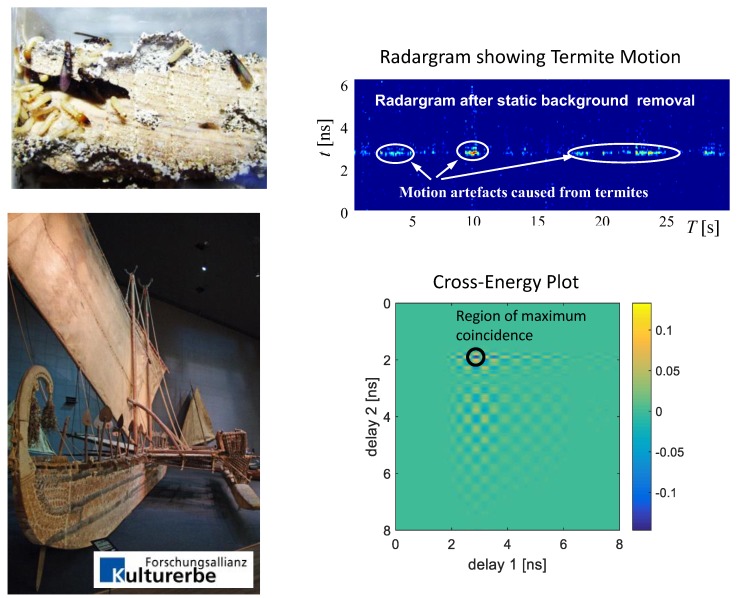
Termite detection in exhibits (courtesy: B. Landsberger, National Museums Berlin).

The final example refers to a situation where the time variations of the scenario are not predictable. In the shown case (see Figure 21), the task was to decide whether or not termites destroy valuable museum exhibits. Termites are hardly visible, because they stay inside the wood. However, they migrate through corridors, which they have eaten into the wood. Hence, there is a good chance to detect their motion and to localize them by high-resolution UWB radar. The roundtrip time where the motion artifacts appear indicates whether the motion comes from inside or outside the wood. If it comes from inside, the wood is affected by wooden pests.

## 5. Conclusions

Microwave imaging is advantageous if the internal structure of optically opaque test scenarios must be investigated. However, in the case of strong multipath scenarios and weak scattering targets, microwave imaging creates challenges for the imaging algorithms and the measurement precision of the scattered field. In order to get reasonable precision, extensive calibrations typically have to be performed with the aim of reducing the systematic errors of the measurement arrangement. However, not infrequently, these calibrations have only limited success because precise calibration standards (covering also the antenna behavior) are missing and/or the reproducibility of the measurements is insufficient.

The situation relaxes if one is interested only in a low number of spatially limited targets, which show some inherently or externally induced time variations of their scattering behavior. One can find or implement such conditions in many tasks of medical microwave imaging, non-destructive testing, law enforcement, and so forth. Due to the time variance of the targets, the perturbing, strong multipath signals and the device clutter may be largely suppressed by respecting only time-variable signal components. Furthermore, the limited spatial extension of the targets reduces the imaging problem to a localization task. Nevertheless, the localization may also be challenging, because multipath components involving the object of interest may appear to be a time-varying target. Such signal components must be excluded prior to target localization.

Microwave localization or imaging requires the observation of the scene from several antenna positions. Due to the time variance of the scenario, the measurements have to be done in parallel with a sufficiently high measurement rate in order to be able to follow the temporal variations of the scenario under testing conditions. This requires UWB devices with many synchronously operating measurement channels and high measurement speed. From the perspective of system cost and measurement speed, the preferable device concepts should be based on sounding signals using sub-nanosecond pulses or wideband pseudo-noise signals, such as M-sequences.

In many practical cases, the targets of interest only weakly scatter the sounding waves. Hence, the noise behavior of the measuring devices becomes a major issue. In the case of UWB devices, two types of randomness are important. These are the randomness of the voltage capturing usually assigned as additive noise (thermal noise, quantization noise) and the randomness of the sampling, also called jitter. While the influence of the additive noise onto the measurement signal may be counteracted by increasing the power of the sounding signal, the impact of jitter does not depend on the signal strength. Jitter provokes additional randomness at steep signal edges. Hence, if there are strong scatterers in the SUT, they will increase the noise level proportional to the level of the sounding signal. Consequently, the detection performance of weak targets will be much worse. Because additive noise and jitter are mainly caused by internal imperfections of the measurement device, one should prefer a device concept that suppresses these random effects as much as possible. As shown in the paper, the pseudo-noise approach outperforms the impulse radar with regard to these aspects. Firstly, the time-extended sounding signal (e.g., M-sequence) provides high signal power for additive noise suppression, even if the signal voltage remains low. Secondly, properly designed M-sequence devices are rigidly synchronized so that any jitter generation may be largely reduced. Finally, the impulse compression (always required in M-sequence devices) spreads the noise provoked by the remaining jitter over the whole impulse response function. Hence, there will no noise elevation at signal edges so that strong static scatterer will not influence the detection of weak time-variable targets.
